# An electrostatic mechanism for Ca^2+^-mediated regulation of gap junction channels

**DOI:** 10.1038/ncomms9770

**Published:** 2016-01-12

**Authors:** Brad C. Bennett, Michael D. Purdy, Kent A. Baker, Chayan Acharya, William E. McIntire, Raymond C. Stevens, Qinghai Zhang, Andrew L. Harris, Ruben Abagyan, Mark Yeager

**Affiliations:** 1 Department of Molecular Physiology and Biological Physics, University of Virginia School of Medicine, Charlottesville, Virginia 22908, USA; 2 Department of Cell and Molecular Biology, The Scripps Research Institute, La Jolla, California 92037, USA; 3 Skaggs School of Pharmacy and Pharmaceutical Sciences, University of California at San Diego, La Jolla, California 92093, USA; 4 Department of Pharmacology, University of Virginia School of Medicine, Charlottesville, Virginia 22908, USA; 5 Departments of Biological Sciences and Chemistry, Bridge Institute, University of Southern California, Los Angeles, California 90089, USA; 6 Department of Integrative Structural and Computational Biology, The Scripps Research Institute, La Jolla, California 92037, USA; 7 Department of Pharmacology, Physiology and Neuroscience, Rutgers New Jersey Medical School, Newark, New Jersey 07103, USA; 8 Center for Membrane Biology, University of Virginia School of Medicine, Charlottesville, Virginia 22908, USA; 9 Cardiovascular Research Center, University of Virginia School of Medicine, Charlottesville, Virginia 22908, USA; 10 Department of Medicine, Division of Cardiovascular Medicine, University of Virginia School of Medicine, Charlottesville, Virginia 22908, USA

## Abstract

Gap junction channels mediate intercellular signalling that is crucial in tissue development, homeostasis and pathologic states such as cardiac arrhythmias, cancer and trauma. To explore the mechanism by which Ca^2+^ blocks intercellular communication during tissue injury, we determined the X-ray crystal structures of the human Cx26 gap junction channel with and without bound Ca^2+^. The two structures were nearly identical, ruling out both a large-scale structural change and a local steric constriction of the pore. Ca^2+^ coordination sites reside at the interfaces between adjacent subunits, near the entrance to the extracellular gap, where local, side chain conformational rearrangements enable Ca^2+^chelation. Computational analysis revealed that Ca^2+^-binding generates a positive electrostatic barrier that substantially inhibits permeation of cations such as K^+^ into the pore. Our results provide structural evidence for a unique mechanism of channel regulation: ionic conduction block via an electrostatic barrier rather than steric occlusion of the channel pore.

## Abstract (web summary)

Intercellular signalling can be mediated by gap junction channels, and calcium blocks this signally during tissue injury. Here, the authors use X-ray crystallography and molecular dynamics to show that the calcium forms an electrostatic barrier to prevent transport of cations.

## Graphical abstract

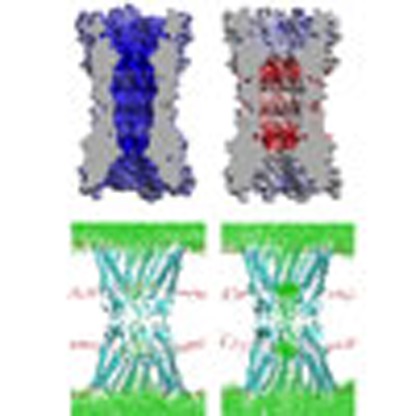

Connexins (Cx) are a 21-member family of integral membrane proteins with four transmembrane (TM) domains (M1–M4), two extracellular loops (E1 and E2), a cytoplasmic (CL) M2–M3 loop and cytoplasmic N- and C-terminal domains (NT and CT; Fig. 1a). Cx monomers assemble as hexameric hemichannels (or connexons) with a central axial pore. Hemichannels from adjacent cells dock to form intercellular, dodecameric gap junction channels (GJCs; Fig. 1b), which can cluster to form gap junction plaques in the plasma membrane (Fig. 1c)^1^,^2^. These dense, quasi-hexagonal arrays enabled electron microscopy of isolated plaques and low-resolution, three-dimensional, image reconstruction^3^. Electron cryomicroscopy (cryoEM) of GJCs assembled from recombinant, human Cx43 at 7.5 (ref. 4) and 5.7 (ref. 5) Å in-plane resolution showed that each hemichannel is comprised of 24 TM α-helices, and the pore has a minimum diameter of ∼15 Å. The extracellular vestibule of GJCs is bounded by a wall of protein, forming a tight seal that excludes extracellular ions and small molecules. An X-ray structure of dodecameric Cx26 at 3.5 Å resolution (Protein Data Bank (PDB) code 2ZW3) revealed that M1 and M2 form the lining of the pore^6^.

GJCs enable direct cell-to-cell exchange of hydrated ions, second messengers and metabolites up to ∼14 Å in the minor dimension^7^,^8^, a function necessary for cell differentiation, growth, cell synchronization and coordinated metabolism. For example, GJCs make the heart a functional syncytium, mediating action potential propagation and ionic conduction between cardiac myocytes, thereby regulating the normal heartbeat and coordinated contraction of the muscle, but also mediating potentially fatal cardiac arrhythmias^9^. GJCs are also responsible for homo- and hetero-cellular communication in vascular endothelial and smooth muscle cells, enabling orchestrated cellular activity over long distances in response to rapidly changing tissue demands^10^.

Owing to their diverse and specialized functions, Cx isoforms have highly variable selectivities, including moderate to significant charge preferences and distinct permeabilities to signalling molecules such as ATP and inositol triphosphate (IP_3_), with a molecular weight (MW) cutoff of about 1,000 Da and a size limit of up to ∼14 Å (refs 11, 12). Many of the processes involving GJCs and hemichannels require proper and tightly controlled regulation, as even modest changes in channel activity or permeability can have a large influence on intercellular signalling and cell viability. A variety of physiological processes and pathological states affect channel activity, including pH^8^,^13^, phosphorylation^10^, membrane lipids^14^, membrane-active agents^15^, membrane potential^16^, trans-junctional voltage (*V*j)^17^, loop-gating^18^,^19^ and intracellular divalent cations^20^ including Ca^2+^ (ref. 21). In some cases, regulation by intracellular Ca^2+^ may involve calmodulin^22^.

In the setting of tissue injury, exemplified by myocardial infarction and stroke, cellular ischaemia impairs membrane integrity, resulting in release of Ca^2+^ from intracellular stores such as the sarcoplasmic reticulum. Ca^2+^ overload during myocardial ischaemia is characterized by a variety of pathological manifestations, including contractile dysfunction, exacerbation of tissue injury and heart failure^23^,^24^. Amelioration of this process represents a potential therapeutic goal for cardiac and cerebral tissue protection during myocardial infarction and stroke. Under normal physiological conditions, ventricular myocytes are electrically coupled by GJCs. Ca^2+^ overload closes GJCs, which likely contributes to the electrical instability and arrhythmias that occur during myocardial ischaemia.

This structural study is focused on the effects of Ca^2+^ on Cx26 (Fig. 1a), which forms homomeric and heteromeric channels (for example, with Cx30 or Cx32) that are permeable to cyclic AMP, IP_3_, glucose and ions with a moderate selectivity for cations^11^,^12^,^25^. Cx26 is involved in hepatic, neural, respiratory, secretory gland, cutaneous and inner ear systems. There are two cochlear gap junction systems (epithelial and connective tissue) in which Cx26 GJCs contribute to K^+^ recycling and glucose homeostasis^26^,^27^. Over 100 Cx26 point mutations have been identified that result in syndromic and non-syndromic deafness in humans. A cluster of deafness-causing mutations is located at the M1/E1 boundary^28^ (Fig. 1a), which coincides with the location of Ca^2+^ coordinating residues identified in this study. Previous experimental studies involving multiple Cx isoforms reveal this segment as uniquely important for loop-gating^29^,^30^,^31^ and regulation by divalent metals^20^.

Low-resolution X-ray scattering^32^, negative-stain electron microscopy^33^ and cryoEM and image analysis^3^ of isolated liver gap junction plaques suggested that Ca^2+^ elicits large-scale conformational changes. Viewed along the channel axis, the sliding and tilting of Cx subunits within each hemichannel was likened to the closure of a camera iris. Indeed, this has been the prevailing model for Ca^2+^-induced GJC gating over the last three decades. Projection images along the pore axis of hemichannels by atomic force microscopy supported the model^34^. The 2ZW3 structure^6^ provided valuable insight into the architecture of the channel but did not reveal a mechanism for regulation by Ca^2+^.

To explore the mechanism by which Ca^2+^ blocks intercellular communication during tissue injury, we determined the X-ray crystal structures of the human Cx26 GJC with and without bound Ca^2+^. Strikingly, the Ca^2+^-bound and Ca^2+^-free channels are nearly identical, ruling out a large-scale steric mechanism for channel block, as well as a local constriction of the pore. Instead, computational analysis suggests that the basis for Ca^2+^-mediated cation exclusion in GJCs is electrostatic in nature and not due to physical occlusion of the pore.

## Results

### Determination of Cx26 GJC X-ray structures

Human Cx26 was expressed in *Sf9* insect cells and purified from enriched membrane preparations (Supplementary Fig. 1). Crystals that displayed isotropic diffraction were grown in the presence or absence of 20 mM Ca^2+^ using a novel facial amphiphile detergent (FA-3)^35^. Both forms crystallized in the *H*32 space group with two monomers in the asymmetric unit (AU; Fig. 2a). Crystals grown with and without Ca^2+^ diffracted to resolutions of 3.3 and 3.8 Å, respectively. We solved the Ca^2+^-bound Cx26 structure independently of the existing Cx26 structure (PDB ID 2ZW3); (ref. 6; see Methods and Supplementary Discussion for details). Molecular replacement (MR) with a dimer of a Cx Cα model based on our cryoEM map (PDB ID 1TXH^5^) resulted in X-ray crystallographic maps of sufficient quality to determine the helix directions and refine the relative orientations of the monomers. A new, extended polyalanine MR model was then built based on the 5.7 in-plane × 19.8 Å cryoEM map from two-dimensional crystals of intact Cx43 GJCs^5^ (Fig. 2b). MR with the hybrid cryoEM/X-ray dimer model and the X-ray crystallographic data resulted in improved initial phases that enabled final model building and refinement (see Methods for details).

Cx26 crystallized as a dodecameric channel, which is aligned along two crystallographic axes. The sixfold axis of the hemichannel is aligned along the crystallographic threefold axis, and the docking interface between hemichannels is oriented along the crystallographic twofold axis. The crystals exhibit type II packing, with the dodecameric channels packed in layers that are separated by approximately the dimensions of one hemichannel (*abc*=75 × 75 × 40 Å^3^; Fig. 2a). There is a single unique crystal contact between dodecamers in adjacent lattice layers. Aided by prime-and-switch phase improvement^36^, nearly all of the amino-acid side chains of the TM and EC domains were eventually accounted for in the electron density maps (Fig. 2c–e). The Ca^2+^-bound Cx26 structure was built and ultimately refined to 3.3 Å resolution (*R*/*R*_free_=0.280/0.313). Although the maps are generally of high quality for the TM α-helices and the extracellular loops, the NT, CL and CT domains could not be modelled because the electron density in those regions is not well defined. The final model consists of residues 19–95 and 134–216 for protomer A and residues 21–95 and 134–213 for protomer B. The Ca^2+^-bound Cx26 structure was used as a search model for MR to solve the structure of Ca^2+^-free Cx26 at 3.8 Å resolution (*R*/*R*_free_=0.301/0.333; Fig. 2h). Crystallographic data, structure refinement and validation statistics are shown in Supplementary Table 1. Although the structures possess superb refinement and validation statistics with reasonable stereochemistry, interpretation of models based on maps at modest resolutions, as is the case here, should be approached with caution.

Analytical size exclusion chromatography (SEC) and multi-angle light scattering indicate that Cx26 purified in FA-3 is a dodecamer (Supplementary Fig. 1a,d), forming a thermally stable (Supplementary Fig. 1b) and homogenous population of defined oligomers (Supplementary Fig. 1c). In addition, there is close similarity between the Cx43 GJC cryoEM map (Fig. 2b) and the X-ray structures (Fig. 2c,e,h). These observations demonstrate that Cx26 crystallized as a *bona fide* GJC.

### Ca^2+^-bound and Ca^2+^-free structures recapitulate the fold of 2ZW3

Although the maximum RMSDs between our structures and 2ZW3 are substantial (Supplementary Fig. 2 and Supplementary Table 2), our independently determined structures recapitulate the general fold of 2ZW3 (ref. 6 and Supplementary Fig. 2). The channel dimensions of the present models are also similar to those reported previously for 2ZW3 (ref. 6): 120 Å from the cytoplasm to the cytoplasm with the pore diameters at the cytoplasmic entrance and the docking interface of 50 and 25 Å, respectively (Fig. 2d). In addition, the docking interactions between hemichannels are very similar in our structures and 2ZW3, and include hydrogen bonding between Q57 residues and between N176 and D179 of apposed hemichannels. The role of the interactions between N176 and D179 in maintaining junctional integrity was recently demonstrated^37^, and is consistent with experiments on docking of compatible Cx isoforms^38^.

### Ca^2+^ is coordinated by adjacent subunits

In the presence of Ca^2+^, *F*_o_–*F*_c_ difference density maps display two discrete 5 *σ* peaks in the AU between subunits at the M1/E1 boundary (green in Fig. 2f,g), corresponding to 6 peaks per hemichannel and 12 peaks per GJC. The difference density peaks reside between the carboxylates of two glutamate residues and the carbonyl oxygen of a glycine, suggestive of a divalent cation-binding site. Ca^2+^ ions were modelled into the difference density peaks, and the coordination distances were consistent with Ca^2+^ binding. The average coordination distance in the refined model (2.6 Å), the coordination number (CN=5) and the formal charge of the site (-2) are consistent with mean values reported in an analysis of nearly 1,500 non-EF hand Ca^2+^-binding proteins for which high-resolution crystal structures were available^39^.

Comparison of the Ca^2+^-bound and Ca^2+^-free electron density maps corroborates the identity of the Ca^2+^ ions: the peaks ascribed to Ca^2+^ in the Ca^2+^-bound Cx26 structure were absent in the Ca^2+^-free *F*_o_–*F*_c_ maps (Fig. 2i,j). The identity of Ca^2+^ ions is therefore supported by (i) the geometries and distances to coordinating oxygen atoms, (ii) the *F*_o_–*F*_c_ (Fig. 2f,g) and *F*_o_–*F*_c_ omit maps, (iii) anomalous difference maps from the Ca^2+^-bound crystals (Supplementary Fig. 3) and (iv) the absence of difference density peaks in the Ca^2+^-free maps (Fig. 2i,j; see Methods for details). Given the identification of the difference peaks as arising from bound Ca^2+^, the sites and Ca^2+^ coordinating residues are shown in Fig. 3a–d. The configuration and interactions of these residues in the Ca^2+^-free structure are shown in Fig. 3e–h.

Ca^2+^ ions in adjacent binding sites are separated by 16 Å, with a 60° rotation around the pore axis. The maximum distance between the Ca^2+^-binding sites across the pore lumen is 23 Å. The Ca^2+^-binding sites coincide with the region of closest approach between the pore-lining M1 helices of adjacent subunits in the hemichannel. However, the major inter-subunit interface is between M1 and M2 (> 500 Å^2^), and includes a salt bridge between E42 and R75, which is not present in the Ca^2+^-free structure, as discussed below. The proximity of the adjacent M1 helices in this region and the substantial M1–M2 contact surface are both conserved in the presence or absence of Ca^2+^ binding (Fig. 3a,e), as well as in 2ZW3 (ref. 6).

Ca^2+^ coordination is mediated by the carboxylate of E47 and the carbonyl oxygen of G45 in one subunit and the carboxylate of E42 in the adjacent subunit (Fig. 3b,d). Thus, it is not surprising that previous sequence-based analyses did not predict this Ca^2+^-binding site as the quaternary structure of the site involves residues in adjacent subunits. The five oxygen atoms that serve as the protein ligands for coordination of the Ca^2+^ ions adopt a square pyramidal geometry, common with CN=5 (ref. 39). Hemispheric coordination of Ca^2+^ leaves the bound ion exposed to the aqueous channel (Fig. 3b), where the Ca^2+^ is expected to remain partially hydrated (as shown by molecular dynamics (MD) simulations discussed below). The carboxylates of the glutamate residues act as coordinating bidentate ligands, with equivalent Ca^2+^ coordination distances and angles between the Oɛ1 and Oɛ2 oxygens.

### The Ca^2+^-bound and Ca^2+^-free structures are nearly identical

Unlike most ion channels, atomic ions are not dehydrated as they pass through GJCs^40^. The pores of the Ca^2+^-bound and Ca^2+^-free channel structures are nearly identical, with a limiting diameter of ∼15 Å (measured between K41 side chains of opposing subunits; Fig. 4a–c and Supplementary Fig. 2d,e). Thus, either channel can easily accommodate fully hydrated K^+^ or Cl^−^ ions as both ions have a first hydration shell diameter of 6.6 Å (Fig. 4c,d). In addition, the Ca^2+^-bound and Ca^2+^-free structures are nearly identical (average Cα RMSD=0.4 Å; Fig. 4a,c). Thus, no major subunit rearrangements are associated with Ca^2+^ binding, and physical occlusion of the pore is not the cause of Ca^2+^-dependent effects on GJC permeability.

### Conformational changes are localized at the Ca^2+^-binding sites

The most significant structural differences between the Ca^2+^-bound and Ca^2+^-free channels occur at the Ca^2+^-binding sites (Fig. 5a–e; Supplementary Fig. 4 and Supplementary Movies 1,2). In the absence of Ca^2+^, the E47 side chain swings by >90^o^ and 7 Å away from the Ca^2+^ coordinating conformation (measured at the Cδ atom) to form an intrasubunit salt bridge with K188, the position of which is essentially invariant between the two structures (compare Fig. 5a,c with b,d). E42 also adopts an alternate conformation in the Ca^2+^-free structure. The coordinating carboxylate of E42 in the Ca^2+^-bound structure rotates ∼90° and forms an intrasubunit hydrogen bond with the Nɛ of R75 (Supplementary Fig. 4). The carbonyl of G45 moves ∼1 Å closer to E42, resulting in an additional intersubunit van der Waals contact in the absence of Ca^2+^. The protein–protein interactions made by E47, E42 and G45 in the Ca^2+^-free structure result in a modest main chain rearrangement relative to the Ca^2+^-bound structure. Specifically, the Ca^2+^-free backbone between residues W44 and Q48 shifts an average of 0.8 Å away from the pore centre and slightly towards M1 of the adjacent subunit.

Among Cx isoforms, most of the native residues are highly conserved in the vicinity of the Ca^2+^-binding site (residues 40–50; Fig. 5f). An acidic residue is conserved in all Cx isoforms at position 47 (Cx26 numbering), whereas an acidic residue at position 42 is absent in 15 of 21 Cx isoforms. However, 11 of these 15 isoforms have an acidic residue (glutamate) at position 41. Therefore, with just a one residue shift in the primary sequence, it is reasonable to speculate that an intersubunit E41/42(B)-G45(A)-E/D47(A) Ca^2+^-binding site is a feature of most GJCs.

### Electrostatic mechanism for Ca^2+^-based regulation of permeation

To investigate whether Ca^2+^ binding significantly alters the electrostatic profile of the channel pore, we calculated surface potentials with (Fig. 6a) and without (Fig. 6b) the contribution of the bound Ca^2+^ ions. For the Ca^2+^-bound structure, the five-coordinate Ca^2+^ ions resulted in an almost entirely positive electrostatic surface potential for the protein lining the pore (Fig. 6a). The Ca^2+^-free pore had a distinctly different electrostatic landscape, characterized by a variegated electrostatic surface with a strongly negative potential in the region of the acidic Ca^2+^-binding residues (Fig. 6b). The significant difference between the Ca^2+^-bound and Ca^2+^-free surface potentials suggests an electrostatic mechanism by which GJCs can regulate ion permeation. The substantial positive electrostatic surface potential in the Ca^2+^-bound structure is particularly informative, as we did not observe physical closure of the channel upon Ca^2+^ binding.

**Figure 1:**
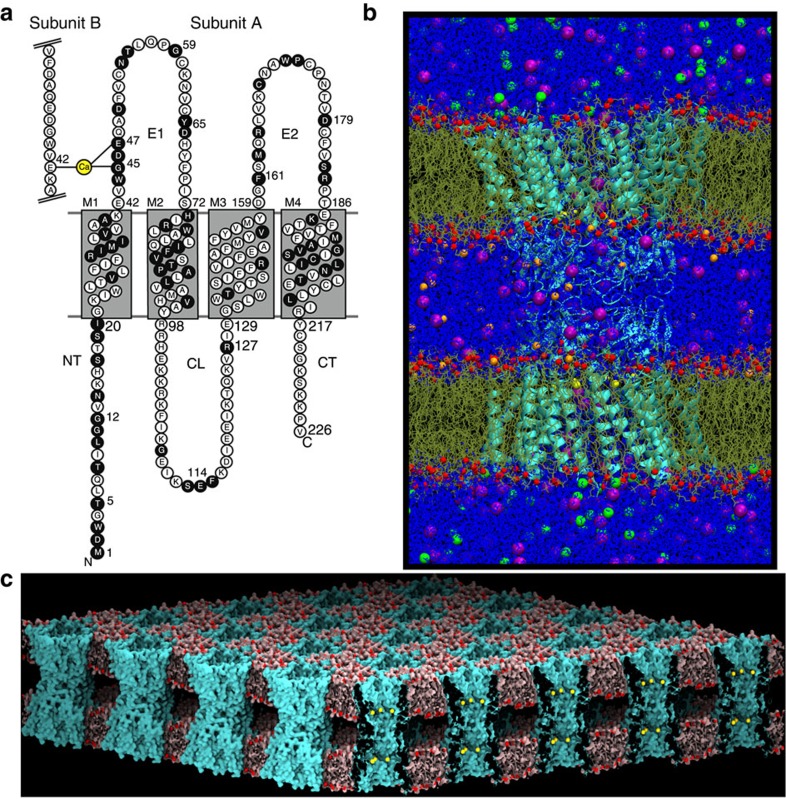
Connexins are the building blocks of dodecameric intercellular gap junction channels. (**a**) All connexins share a similar topology, with four transmembrane α-helices (M1–M4), two extracellular loops (E1 and E2) and three cytoplasmic domains: the N-terminal tail (NT), M2–M3 cytoplasmic loop (CL) and the carboxy-tail (CT). Over 100 point and deletion mutants have been identified in Cx26 that are linked to deafness. Sixty-nine of these mutants have been tested for trafficking and functionality and are mapped onto the Cx26 topology diagram, indicated by circles coloured solid black with white lettering. Shown at the upper left are the specific residues from adjacent subunits (E42 in subunit B and E47 and G45 in subunit A) identified in this study that coordinate Ca^2+^ (yellow circle). (**b**) The gap junction channel (cyan) as derived from the Ca^2+^-bound crystal structure reported here is comprised of two hexameric Cx hemichannels. The hemichannels dock end-to-end to form an intercellular channel that spans the membrane bilayers of apposed cells and the extracellular gap, from which the name is derived. The phosphorous atoms (red spheres) in the lipid head groups delimit the membrane bilayer with internal aliphatic lipid chains (gold). The ions, depicted as spheres sized by their relative ionic radii, are K^+^ (green), Cl^−^ (purple) and Na^+^ (orange). As revealed in this study, the Ca^2+^ ions (yellow) bind to residues at the interface between the membrane and extracellular gap. The NT, CL and CT of each Cx subunit are not shown, as their densities were not well defined in the map. (**c**) Representation of a gap junction plaque comprised of channels that pack with quasi-hexagonal symmetry. The cross-section at the right enables visualization of the intercellular pore and the location of bound Ca^2+^ ions at the interface between the transmembrane α-helices and the extracellular gap. Protein, ions and the membrane bilayers are colored as in **b**.

To determine whether the electrostatic surface potentials affect ion permeation and selectivity, we performed MD simulations of the Ca^2+^-bound (Fig. 6c) and Ca^2+^-free (Fig. 6d) X-ray structures embedded in two 1-palmitoyl-2-oleoyl-*sn*-glycero-3-phosphocholine (POPC) lipid bilayers. Each system included explicit water molecules and was neutralized and ionized with 140 mM KCl in the cytoplasmic region and 140 mM NaCl in the extracellular region (Fig. 1b), resulting in a total system size of ∼300,000 atoms. For the purposes of electroneutrality, the Cl^−^ concentration was set to 140 mM in the MD calculations. However, we realize that the physiologic cytoplasmic concentration of Cl^−^ is ∼4 mM, with most of the additional negative charge contributed by cytoplasmic proteins. Simulations consisted of unrestrained equilibration (20 ns) followed by a production phase (at least 30 ns; Supplementary Fig. 5). For this analysis, we defined Ca^2+^ coordination as a distance of <2.6 Å between a coordinating atom and the Ca^2+^ ion.

In the Ca^2+^-bound simulation (Fig. 6c and Supplementary Movie 3), Ca^2+^ ions remained bound, and coordination by the E42 and E47 carboxylates (E42-Oɛ and E47-Oɛ) was stable (Supplementary Figs 6,7), with either three or four of the carboxylate oxygens coordinating the Ca^2+^ at all times. However, Ca^2+^ coordination by the carbonyl oxygen of G45 was variable. Depending on the site, coordination by the carbonyl oxygen was maintained throughout, lost or lost and then restored (Supplementary Movie 4). Participation in Ca^2+^ coordination by G45-O was stable at only one site during the simulation (Supplementary Movie 5). We suspect the loss of Ca^2+^ coordination by G45-O in the MD simulations may be due to known limitations in the nonpolarizable force field describing the interactions between divalent cations and carbonyl oxygen atoms^41^. To assess whether loss of G45-O Ca^2+^ coordination altered the macroscopic Cx26 channel properties, we repeated the MD simulation with restraints on the distances between the G45-O atoms and the Ca^2+^ ions (Supplementary Figs 8 and 9), which displayed Ca^2+^ coordination geometry similar to that of the Ca^2+^-bound X-ray structure. Specifically, Ca^2+^ coordination by G45-O and all four E42 and E47 carboxylate oxygens was stable at most sites for the majority of the MD simulation, with occasional excursions by a single carboxylate oxygen, which reduced the number of protein ligands to four. Importantly, MD simulations with restraints on the distance between Ca^2+^ and G45-O did not alter the cationic permeability results without restraints (compare Fig. 6c and Supplementary Fig. 8b). Throughout the simulations, three or four water molecules participated in ion coordination at each Ca^2+^-binding site, maintaining a total coordination number of seven to eight (Fig. 6e,f).

A notable result of the simulations was the difference in K^+^ ion occupancy within the pore between the Ca^2+^-bound and Ca^2+^-free channels. For the Ca^2+^-bound channel (Fig. 6c and Supplementary Movie 3), K^+^ ions were almost completely excluded from the pore. In the Ca^2+^-free simulation (Fig. 6d and Supplementary Movie 6), K^+^ ions rapidly entered the channel and clustered around the Ca^2+^-binding sites. We calculated the potential of mean force (PMF) for K^+^ and Cl^−^ along the Ca^2+^-bound and Ca^2+^-free channel axes based on ion density distributions in the all-atom simulations. The resultant K^+^ and Cl^−^ free energy profiles (Supplementary Fig. 10) paired with the electrostatic analysis suggest a novel mechanism for Ca^2+^-dependent ion selectivity, in which Ca^2+^ binding functions as an electrostatic switch that dramatically reduces cation permeability.

## Discussion

We report here the first structural identification of Ca^2+^-binding sites in GJCs. The X-ray structures of Cx26 GJC in the presence and absence of Ca^2+^ revealed that Ca^2+^ is coordinated at the M1/E1 boundary by residues from adjacent subunits. We expect that the Ca^2+^-binding sites identified here are likely the same as in undocked, functional Cx26 hemichannels, which are also regulated by Ca^2+^. Ca^2+^ binding to the Cx26 GJC did not trigger a global conformational change to a sterically closed state, as observed in low-resolution studies of Cx GJCs^3^ and hemichannels^34^. As Cx26 was co-crystallized with Ca^2+^, we assume that large-scale structural rearrangements were possible and would have occurred before crystallization. However, we observed only local conformational changes in the vicinity of Ca^2+^ coordination, which did not occlude the pore. Likewise, the side chain conformational changes occur for residues adjacent to the lumen of the pore rather than on the perimeter. Therefore, it is unlikely that the observed conformational changes are due to crystal packing artefacts.

Electrostatic calculations and MD simulations revealed that block of K^+^ permeation is achieved by the extensive positive surface potential conferred by the bound Ca^2+^; that is, Ca^2+^-binding creates an electrostatic barrier that impedes cation entry into the pore. We propose that Ca^2+^ binding in GJCs acts as an electrostatic switch that dramatically shifts the charge selectivity of the channel, creating a strongly positive environment within the pore that inhibits the ability of cations such as K^+^ to enter and thereby permeate the pore. Given the sequence conservation of residues that participate in Ca^2+^ binding, we expect that the electrostatic switch regulating Ca^2+^-dependent ion selectivity is a general mechanism in the family of Cx junctional channels.

In biomedical terms, this study of junctional channels is relevant to conditions of Ca^2+^ overload, which occur in tissues subject to injury. It is well known that pathologically elevated cytosolic Ca^2+^ levels cause inhibition of gap junction coupling, reducing the intercellular propagation of toxic signals from dying/diseased cells. It is generally believed that this occurs at Ca^2+^ levels above micromolar^42^,^43^,^44^. At lower concentrations, there is evidence that Ca^2+^ can permeate junctional channels^44^,^45^,^46^. Our structural and computational results suggest that Ca^2+^ binding within GJCs profoundly alters the character of permeating species. Due to the positive electrostatic potentials within the pore that is induced by Ca^2+^-binding, we expect that the channels will not only become far less permeable to cations but also any molecules with net positive charge.

There is theoretical and electrophysiological evidence that barriers to the flux of molecules and ions can be explained by purely electrostatic effects, without a requirement for steric occlusion^47^,^48^. Our results provide the first structural evidence for a unique mechanism of channel regulation: block of ionic conduction via an electrostatic barrier rather than steric occlusion of the pore.

## Methods

### Expression, solubilization and purification of human Cx26 for crystallization

Expression of human Cx26 C211S, C218S (in which two cysteine residues in the CT were replaced with serine to prevent non-native disulfide bond formation) with a C-terminal hexahistidine tag was performed by infection of *Spodoptera frugiperda* (*Sf9*) insect cells with recombinant baculovirus. Cells were infected at a multiplicity of infection (MOI) of 5 and harvested 48–52 h post infection. All subsequent steps were performed at 4 °C, unless otherwise indicated. Cells were lysed by 20–25 strokes using a Dounce homogenizer in low-salt buffer (50 mM HEPES, pH 7.5, 50 mM NaCl, 0.5 mM EDTA and 1 × Complete protease inhibitor cocktail (Roche)). Nucleic acids were digested by adding 10 units of Benzonase (EMD Biosciences), adjusting the buffer to include 2.5 mM MgCl_2_, followed by 15 min of gentle rocking. The lysate was clarified by ultracentrifugation (100 k × *g*, 30 min, Beckman model Optima LE-80K), and the pellet was washed with low-salt buffer, high-salt buffer (low-salt buffer with NaCl increased to 1 M) and final wash buffer (high-salt buffer without EDTA). The final pellet contained enriched plasma and organellar membranes. Membranes were stored as aliquots, flash frozen in liquid N_2_ and stored at −80 °C until use.

Membranes (∼5 g wet weight) were thawed on ice and resuspended in 50 ml extraction buffer (50 mM HEPES, pH 7.5, 300 mM NaCl, 10 mM imidazole, 2% (v/v) glycerol and 1 × Protease Inhibitor Set V (Calbiochem)). Membranes were disrupted by ten strokes using a Dounce homogenizer in order to obtain a small particle size for efficient solubilization. A 10% (w/v) stock solution of *N*-decyl-β-D-maltopyranoside (DM) detergent (Anatrace) was added to the resuspended membranes to a final concentration of 1% (w/v; ∼1 mM; ∼10 × critical micelle concentration (CMC)). Membranes were solubilized for 3 h on a rotator and then clarified by ultracentrifugation (150 k × *g*, 40 min, Beckman model Optima LE-80K). After detergent solubilization, it was found that maintaining the protein in buffers with high ionic strength (0.5–1.0 M NaCl) was essential to ensure protein stability and solubility during affinity purification and subsequent concentration steps. His-tagged Cx26 was purified from solubilized membranes by binding the protein to immobilized cobalt affinity resin (Clontech) at a ratio of 1 ml affinity resin per 50 ml detergent extract. After batch binding for 30–60 min, the slurry was poured into a column, and the beads were washed with at least 5 column volumes (CVs) of extraction buffer with 1% (w/v) DM, followed by 5 CVs of wash buffer (50 mM HEPES, pH 7.5, 1 M NaCl, 0.5% (w/v) DM, 20 mM imidazole and 1 × Protease Inhibitor Set V). DM was exchanged for FA-3 on the column by addition of two CVs of exchange buffer (50 mM HEPES, pH 7.5, 1 M NaCl, 20 mM imidazole, 2.5% (v/v) glycerol and 1 × Protease Inhibitor Set V) containing 0.08% (w/v) FA-3 detergent (4 × CMC) to the resin, and 1 CV was allowed to flow through the column. The column was capped, kept stationary and incubated with the new detergent for 15 min. The resin was further washed with 1 CV of FA-3 exchange buffer. Cx26 was then eluted from the column in 1 ml fractions with elution buffer (exchange buffer containing 250 mM imidazole) containing 0.05% FA-3. Fractions with the highest protein concentrations, as estimated by absorption at A_280_ (NanoDrop 1000 spectrophotometer; Thermo Scientific), were pooled and concentrated to ∼0.5 ml in a 100-kDa molecular weight cutoff (MWCO) concentrator (Millipore). Imidazole was removed using a G-25 desalting column (GE Healthcare) equilibrated in final buffer (100 mM HEPES, pH 7, 1.0 M NaCl, 2.5% (v/v) glycerol and 1 × Protease Inhibitor Set V) containing 0.02% (w/v) FA-3. For crystallization trials, the eluate from the desalting column (∼1 ml) was concentrated to 4–6 mg ml^−1^ (150–200 μM) using a 500-μl or 4-ml concentrator (100 kDa MWCO; Millipore).

### Crystallization of human Cx26

Crystals of Cx26 in FA-3 could be grown reproducibly at 25 °C using hanging-drop or sitting-drop vapour diffusion methods by mixing 1 ml of Cx26 in final buffer with 0.02% FA-3 and 1 μl of a reservoir composed of 100 mM HEPES, pH 7, 15 mM sodium formate, 20 mM CaCl_2_ and 12% PEG 3350. Crystals for X-ray diffraction experiments were on average 50 × 50 × 25 μm^3^ and typically grew within 1–2 weeks in the presence of 5–50 mM CaCl_2_. The best diffracting Ca^2+^-bound crystals were grown from setups with 20 mM CaCl_2_. To grow Ca^2+^-free Cx26 crystals, the same crystallization setup and reservoir buffer were used, with the CaCl_2_ omitted from the formulation. In the absence of CaCl_2_, crystals required at least a month to grow. Cryoprotectant (either glycerol or ethylene glycol) in harvesting buffer (100 mM HEPES, pH 7, 20 mM sodium formate, 25 mM CaCl_2_ (omitted when harvesting Ca^2+^-free crystals), 14% PEG3350 and 0.02% FA-3) was added directly to the crystallization drop by increasing the concentration of glycerol or ethylene glycol in the crystallization drop in 5% increments every 5–10 min. Crystals were then harvested directly from the drop into 50–100 μm diameter loops (Hampton Research), flash frozen by plunging into liquid N_2_ and loaded into ALS-style pucks.

### Synchrotron X-ray data collection

For the Ca^2+^-bound Cx26 crystals, X-ray diffraction data were collected at the SER-CAT 22-ID beam line at the Advanced Photon Source at Argonne National Laboratory. Initial indexing of the crystals by HKL2000 (ref. 49) suggested a rhombohedral Bravais lattice (*H*3), a maximal diffraction resolution of 3.0 Å and a mosaicity of 0.5°. Using a 50-μm beam and 1° oscillations, several wedges of data were collected from one crystal. Ultimately, three data sets were integrated separately and merged upon scaling, with an overall *R*_merge_ of 7.5% and a resolution cutoff of 3.1 Å.

For the Ca^2+^-free Cx26 crystals, X-ray diffraction data were collected at the GM/CA-CAT 23-ID beam line at the Advanced Photon Source. The Ca^2+^-free crystals had the same Bravais lattice and were isomorphous with the Ca^2+^-bound crystals, although reflections could only be observed to 3.6 Å resolution. Two wedges of data from one crystal were merged upon scaling, with an overall *R*_merge_ of 7.5% and a resolution cutoff of 3.8 Å. Both crystal forms (Ca^2+^-bound and Ca^2+^-free) conformed to the *H*32 space group with two molecules in the AU with a Matthews coefficient (*V*_m_) of 3.7 and a solvent content of 67%. Crystallographic statistics are listed in Supplementary Table 1. Resolution limits for the data sets were determined based on data completeness, I/s, and CC^1/2^ (ref. 50) in the highest resolution shells. For the Ca^2+^-free data sets, a strong solvent pattern in the diffraction images at ∼3.5 Å resolution prevented inclusion of data beyond 3.6 Å resolution. Resolution limits for model refinement and validation were determined based on the CC^1/2^ values and assessment of the quality of the electron density map.

### Structure determination

We solved the Ca^2+^-bound Cx26 X-ray crystallographic structure first using the previously determined Cx26 structure (PDB ID: 2ZW3)^6^ as the MR search model. We also solved the structure independently using a MR model based on a 5.7 × 19.8 Å^2^ resolution cryoEM map derived by analysis of two-dimensional crystals of Cx43 (ref. 5). These two structure determination methods yielded the same solution. An explanation of our reasons for solving the structure independently is included in the Supplementary Discussion. The Ca^2+^-bound structure presented here is the result of MR with the cryoEM model. Using Molrep^51^ in CCP4 (ref. 52) and Phaser^53^ in Phenix^54^, we performed MR searches for multiple copies of a Cx26 monomer composed of just the four TM domains, which consisted of 87 carbon atoms. These searches failed, but a search with a dimer of the cryoEM based Cα model (PDB ID: 1TXH^5^) succeeded and revealed two monomers in the AU of the *H*32 crystals. The resultant maps had clear 2*F*_o_–*F*_c_ electron density, corresponding to the TM domains of the Cx26 GJC that extended beyond the limits of the 1TXH Cα model. The biological sixfold axis that gives rise to the Cx26 hemichannel was coincident with the crystallographic threefold axis, and the biological twofold axis that gives rise to the GJC (at the interface between hemichannels) was coincident with the crystallographic twofold. We converted the Cα model to polyalanine (polyAla) in Coot^55^ and performed rigid body refinement in Phenix. Further refinement resulted in electron density maps of sufficient quality in the TM domain to determine the directionality of the TM α-helices. We reversed the direction of the M2 helix in the 1TXH model and generated new monomeric and dimeric models consisting of ideal polyAla α-helices. We extended this model through rounds of manual building in Coot, refinement in Phenix and density modification and non-crystallographic symmetry (NCS) averaging with RESOLVE^36^. Although new electron density appeared that could be attributed to polypeptide, the maps did not improve sufficiently to allow additional model building.

We then used the cryoEM map^5^ to construct a new model beginning with a dimer of the 4-TM bundle, represented as polyAla. Pymol^56^ was used to manually fit the dimeric polyAla model consisting of 174 residues into the cryoEM map. On the basis of secondary structure predictions and the cryoEM density map, we placed two ideal polyAla α-helices and two β-strands in the extracellular region. We then modelled four additional, polyAla α-helices as cytoplasmic extensions of the TM domains, based on the cryoEM map contoured at 1-*σ*. The final monomeric model based on the cryoEM map consisted of 184 residues. We generated a dimer of the expanded model by application of the NCS operators determined from the dimeric 4-TM polyalanine model that we built with the aid of the initial X-ray crystallographic maps. It was important to use the NCS operators based on the X-ray crystallographic map to generate the dimer because the relative orientations of the Cx subunits differed slightly between the two- and three-dimensional crystals. The expanded dimeric model consisted of 368 residues.

**Figure 2:**
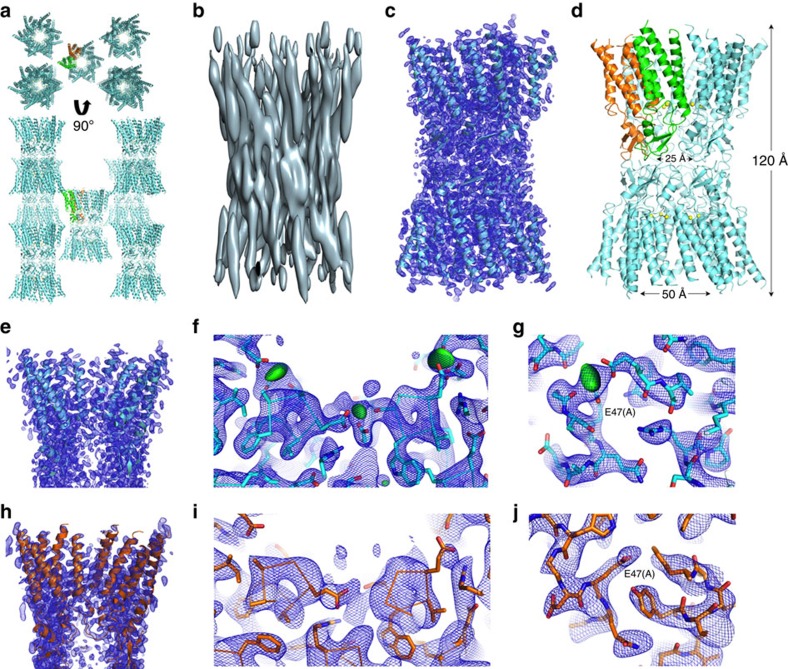
Crystal structures of Ca^2+^-bound and Ca^2+^-free Cx26 gap junction channels. (**a**) Packing of Cx26 dodecamers (cyan) in the *H*32 lattice. The main crystal contact involves residues on the cytoplasmic surface of the channel. The two subunits in the asymmetric unit, a dimer of Cx26, are shown in green and orange. (**b**) Three-dimensional cryoEM map at 5.7 Å resolution in *X*, *Y* and 19.8 Å resolution in *Z* derived from electron cryocrystallography of two-dimensional crystals of human Cx43 channels in gap junction plaques^5^. (**c**) Ca^2+^-bound Cx26 structure (cyan) and the 2*F*_o_−*F*_c_ electron density map (blue mesh) at 3.3 Å resolution and contoured at 1.5-*σ*. (**d**) Side view of the dodecameric assembly of Cx26 forming the GJC. Ca^2+^ atoms are shown as yellow spheres. Distances are measured between Cα atoms. (**e**) A cross-sectional view of the Ca^2+^-bound structure (cyan) and map (blue mesh) showing the funnel-shaped cytoplasmic region of the pore that narrows in proceeding to the extracellular vestibule. (**f**) Inter-subunit residues that form the Ca^2+^-binding sites, as revealed by the 3.3 Å resolution crystal structure. The difference density peaks (the *F*_o_−*F*_c_ maps) indicate the unrefined positions of Ca^2+^ ions (green). Three binding sites are shown in this cytoplasmic view. (**g**) A close-up of one of the Ca^2+^-binding sites shown in **f**. (**h**) A cross-sectional view of the Ca^2+^-free structure (orange), shown with a 3.8 Å resolution 2*F*_o_–*F*_c_ map (blue mesh). (**i**) A view of the Ca^2+^-free structure as shown in **f** for the Ca^2+^-bound structure, showing both 2*F*_o_−*F*_c_ and *F*_o_−*F*_c_ maps. Note the absence of any green difference density peaks in the *F*_o_–*F*_c_ map. (**j**) A close-up of E47 in subunit A for the Ca^2+^-free structure. To better visualize the conformational change of E47, the molecule has been rotated significantly compared with the perspectives shown in **g** and **i**. Side chains are shown as sticks, with atoms coloured as: carbon, cyan (Ca^2+^ bound) or orange (Ca^2+^ free); oxygen, red; nitrogen, blue. The 2*F*_o_−*F*_c_ maps (blue mesh) and the *F*_o_–*F*_c_ maps (green) are contoured at 1.5-*σ* and 4-*σ*, respectively.

**Figure 3:**
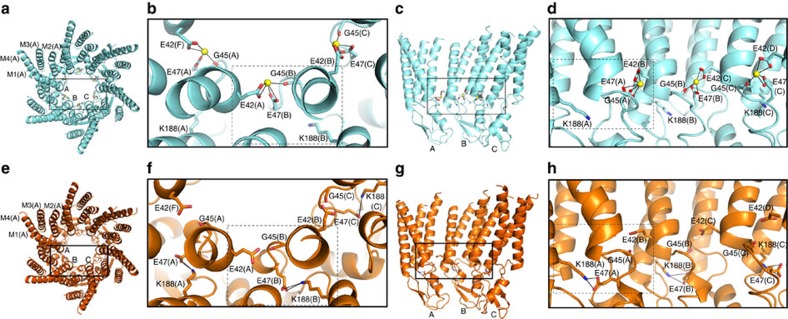
The binding sites for Ca^2+^ in gap junction channels are positioned at the subunit–subunit interface. The Ca^2+^-bound (cyan) and Ca^2+^-free (orange) structures, as well as the Ca^2+^ ions (yellow) are coloured identically to Fig. 2. (**a**,**e**) Cytoplasmic, axial view of the (**a**) Ca^2+^-bound and (**e**) Ca^2+^-free hemichannel, with three subunits (A, B and C) showing labels for TM helices M1–M4 in subunit A. (**b**,**f**) Close-up, cytoplasmic views of Ca^2+^ coordination sites in the (**b**) Ca^2+^-bound and (**f**) Ca^2+^-free structures. The dashed boxes delimit the residues involved in coordination of a single Ca^2+^ ion. (**b**) and their interactions in the absence of Ca^2+^ (**f**). The Ca^2+^ atoms are bound between adjacent subunits, utilizing a pentamer of protein ligands, and exhibit hemispheric coordination, with the unliganded face exposed to bulk solvent in the pore of the channel. (**c**,**g**) Side views of the (**c**) Ca^2+^-bound and (**g**) Ca^2+^-free hemichannels. For clarity, the three subunits nearest to the viewer have been removed. (**d**,**h**) Close-up, side views of the Ca^2+^ coordination sites in the (**d**) Ca^2+^-bound and (**h**) Ca^2+^-free structures. The dashed boxes delimit the residues involved in coordination of a single Ca^2+^ ion (**d**) and their interactions in the absence of Ca^2+^ (**h**). Ca^2+^ coordination and hydrogen bonding are shown as solid black lines.

**Figure 4:**
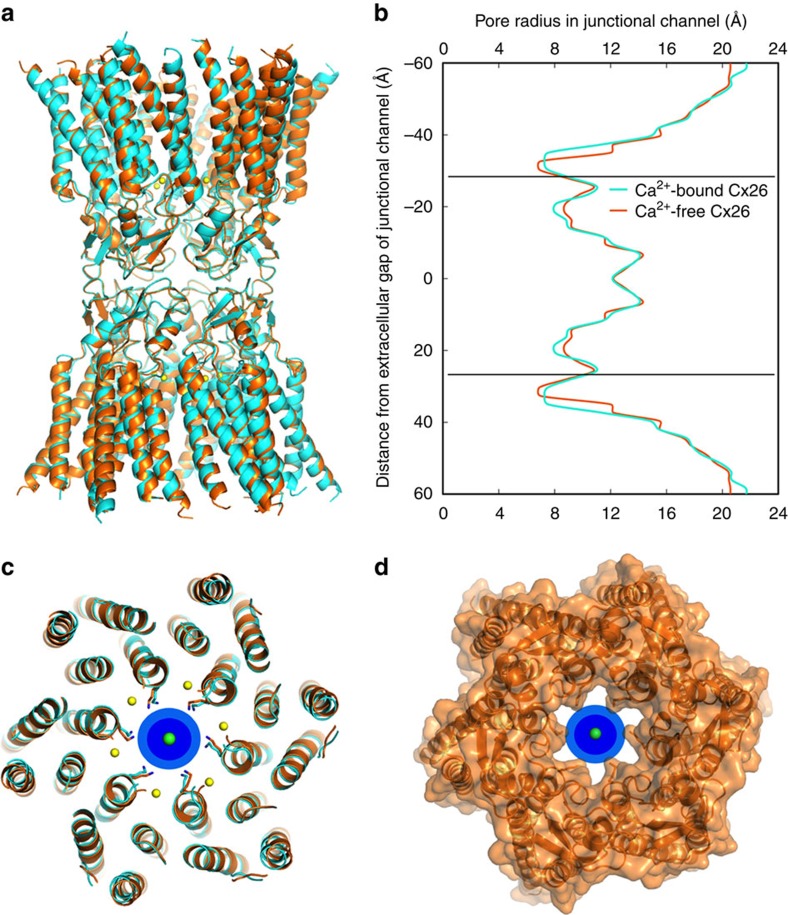
The Ca^2+^-bound and Ca^2+^-free structures are nearly identical, with the same pore diameter. The Ca^2+^-bound (cyan) and Ca^2+^-free (orange) structures, as well as the Ca^2+^ ions (yellow) are coloured identically to Figs 2 and 3. (**a**) Side views of superimposed Ca^2+^-bound and Ca^2+^-free GJC structures. (**b**) Plots of distance from the centre of the extracellular gap versus pore radius for the Ca^2+^-bound channel (cyan line) and the Ca^2+^-free channel (orange line). The solid horizontal lines at ±28 Å indicate the locations of the Ca^2+^-binding sites within each hemichannel. (**c**) Overlay of the Ca^2+^-bound (cyan) and Ca^2+^-free (orange) hemichannels, viewed from the extracellular gap and shown at the level of the minimum pore diameter (∼15 Å, as measured between Nζ atoms of K41) within the hemichannel, located between K41 residues of subunits that are directly across from one another. The side chains for the K41 residues are shown as sticks. A K^+^ ion is shown at the centre of the hemichannel with its first (7 Å diameter) and second (12 Å diameter) hydration shells shown in dark and light blue, respectively. (**d**) A van der Waals surface representation of the Ca^2+^-free Cx26 hemichannel. The K^+^ ion and hydration spheres are shown as in **c**.

We used Phaser-MR within Phenix^53^ for MR, with the expanded dimer as the search model and the *H*32 X-ray crystallographic data as the target. The search included data from 35 to 3.5 Å resolution and succeeded with a top translation function Z-score of 6.9. Rigid body refinement of the MR model in Phenix yielded crystallographic *R* and *R*_free_ values of 0.511 and 0.526, respectively, and 2*F*_o_–*F*_c_ maps clearly showed density for the full dodecameric channel (Fig. 2c).

We used Coot to rebuild the model based on the X-ray crystallographic maps, removing all but two turns of one α-helix from the EC domain because of poor density and disagreement between the model and maps in that region. We also made contiguous α-helices for the TM domains from the helical fragments used for MR and rigid body refinement. After rounds of real space modification of the polyAla model and refinement, followed by prime-and-switch density modification with RESOLVE^36^, we were able to identify and position aromatic side chains and disulfides, which enabled assignment of the sequence and the TM α-helices. We first identified M2 by electron density that we determined was the sequence HYFPxxH, corresponding to the Cx26 sequence HYFPISH (residues 67–73) leading into M2 from E1 within the extracellular domain. Next, we identified M3 by density for a polypeptide with the sequence FF(long)xxF (where ‘long' indicates an extended side chain, corresponding to the Cx26 sequence FFRVIF in the centre of M3 (residues 141–146)). Then, we defined M4 by density for a sequence KxxFxxF, corresponding to the Cx26 sequence KTVFTVF (residues 188–194) at the extracellular end of M4, near the end of E2 in the extracellular domain. Finally, we confirmed the identity of M1 by density for a sequence WxxxxFxF, corresponding to the Cx26 sequence WLTVLFIF (residues 24–31) in the cytoplasmic half of M1, near the NT domain. The overall map quality, including that for the extracellular domain, continued to improve as we added side chains. We began building the backbone of E2, connecting M3 and M4. Then, based on strong density for a disulfide (there are three in the EC domain) and nearby recognizable density for a tryptophan and other bulky side chains, we built side chains for the Cx26 sequence MQRLVKCNAWPCP (residues 163–175). We continued adding sequence in this region based on a second disulfide (between C58 and C180) and a neighbouring phenylalanine (F181). During building and refinement, the maps improved significantly with the appearance of a missing stretch of 14 residues (S162–P175) in E2 of the extracellular domain. The new density revealed a β-strand that, with an existing β-strand, formed a β-hairpin at the extracellular gap (extending from L166–F181). Finally, we were able to complete the model of the TM and EC domains, consisting of residues 18–93 and 135–216 in molecule A (156 of 226 total) and 21–96 and 134–213 in molecule B (154 of 226). Therefore, ∼70% of the total sequence is accounted for in the final model.

Throughout building and refinement, several strong positive *F*_o_–*F*_c_ difference density peaks were present that were not accounted for by the protein model, exemplified by the peak in Supplementary Fig. 3a (denoted by ‘?'), which may be a detergent molecule. Two NCS-equivalent peaks in the AU that were greater than 5-*σ* were located at an interface between Cx subunits, at the end of M1, extending into the start of E1. The peaks localized near the carboxylates of a glutamate from one Cx (E42) and the carboxylates of a second glutamate (E47) and the carbonyl oxygen of a glycine (G45) from the adjacent Cx subunit (Fig. 2e–g). The difference density surrounded by four carboxylates and one carbonyl was strongly suggestive of divalent cation coordination. As the crystals had been grown in the presence of 20 mM CaCl_2_, we modelled Ca^2+^ ions into the *F*_o_–*F*_c_ density and refined the model with and without Ca^2+^ coordination restraints in Phenix. Even in the absence of the Ca^2+^ restraints, the model refined well with the added Ca^2+^ ions, accounting for the *F*_o_–*F*_c_ density and with appropriate distances to the coordinating oxygens.

To help verify the presence of Ca^2+^ at these sites, we merged the data sets from three different crystals grown in CaCl_2_ (in order to amplify the weak Ca^2+^ anomalous signal) and calculated an anomalous difference map at 4 Å resolution using Ca^2+^-free, model phases. The only peak observable at >4-*σ* in the anomalous map partially overlapped with one of the *F*_o_–*F*_c_ difference density peaks (+5-*σ*) that had guided the placement of a Ca^2+^ atom (Supplementary Fig. 3). Although not definitive or necessarily coincident, the overlap of anomalous and difference peaks provides some evidence of a metal-binding site. Significantly, after refinement, there was an absence of negative *F*_o_–*F*_c_ density at the binding site and even some residual positive density on the side of the coordinated ion exposed to the pore. Removal of Ca^2+^ ions from the model, subsequent refinement and calculation of omit maps gave rise to the re-appearance of >4-*σ F*_o_–*F*_c_ peaks at the site where Ca^2+^ ions had been modelled. In addition, anomalous difference maps were calculated, and a >4-*σ* peak was present at one of the Ca^2+^-binding sites in the AU (Supplementary Fig. 3). After modelling the Ca^2+^ ions and subsequent refinement, the binding sites were analysed for agreement with the published literature and databases of Ca^2+^-binding sites^39^,^57^. The coordination number (*n*=5), formal charge of the site (*n*=−2) and coordination bonding distances (both average side chain carboxylate distance and average main chain carbonyl distance=2.6 Å) were all within the reported ranges for Ca^2+^ binding. Although the resolution limit of the diffraction data precluded the definitive placement of water molecules, it is suspected that the residual positive density was likely due to the presence of coordinating water molecules. Water coordination is expected given the hemispheric and incomplete coordination of the Ca^2+^ by the protein. MD simulations of the Ca^2+^-bound channel also showed hydration of the bound Ca^2+^ ions (Fig. 6c,d).

Electron density for additional turns of α-helices was present on the cytoplasmic side of the channel, but the density was not sufficiently well defined to enable modelling of the CL or either of the termini. Both the CL and CT have been shown to be involved in GJC regulation^58^ and to interact with one another under acidic conditions^59^. In our crystals grown at pH 7, we suspect that the CT is involved in the primary crystal contact, found near the ends of the M4 helices of adjacent dodecamers in the crystal lattice (Fig. 2a). We note that this crystal contact only occurs at every other subunit.

We solved the Ca^2+^-free structure by MR with the Ca^2+^-bound structure, after removal of the Ca^2+^ ions. The Ca^2+^-free crystals were isomorphous with the Ca^2+^-bound crystals, and the initial maps after MR and refinement were of good quality for the TM and extracellular domains. Despite the lower resolution limit of the diffraction data (3.8 Å), prime-and-switch NCS averaging and density modification resulted in maps that allowed us to obtain a well-refined model of the Ca^2+^-free GJC. Significantly, no peaks in either *F*_o_–*F*_c_ or anomalous maps appeared in the vicinity of the Ca^2+^-binding site identified in the Ca^2+^-bound structure.

### MD simulations

We used VMD 1.9.1 (ref. 60) to build systems comprised of full Cx26 GJCs embedded in two POPC bilayers. We constructed each system by hydrating the protein with Solvate 1.0.1 (ref. 61) and then removed the water molecules in the surrounding lipid-facing portion of the protein based on hydrophobicity and the positions of lipid-facing, aromatic amino acids. We used the VMD Membrane Builder plugin to generate lipid bilayers for use with the CHARMM36 force field^62^. We manually positioned the bilayers relative to the full GJC. Then, we removed lipids overlapping with the protein and within the channel. To generate the water box for the system, we used the VMD plugin Solvate. Finally, we neutralized and ionized the system using the VMD plugin Autoionize. Last, we selectively removed ions from the parts of the system corresponding to the cytoplasmic or extracellular spaces and then repeated neutralization and ionization in order to ionize the cytoplasmic space with 140 mM KCl and the extracellular space with 140 mM NaCl. In the case of the Ca^2+^-free channel, we used the same method to add intracellular CaCl_2_, in order to make the total Ca^2+^ equivalent in the simulations. (No cytoplasmic Ca^2+^ was added to the simulations of the Ca^2+^-bound channel.)

We used the CUDA-enabled version of NAMD 2.9 to perform the equilibrium MD simulations of these systems using the CHARMM36 force field for the POPC bilayers^62^ and the CHARMM27 (ref. 63) force field for all other atoms, employing the CMAP correction for handling dihedral angles in polypeptides. Admittedly, there are inherent limitations in attempting to simulate the behaviour of metals covalently bound to proteins, mainly due to the lack of well-established and accurate parameters to describe protein–metal coordination in any of the available MD force fields^41^.

For each simulation, minimization and equilibration followed the procedure described at http:// www.ks.uiuc.edu/Training/Tutorials/science/membrane/mem-tutorial.pdf. We first equilibrated the lipid acyl chains, keeping the rest of the system fixed. Next, we restrained the protein in order to first minimize and equilibrate the rest of the atoms of the system. Then, we released the restraints on the protein and equilibrated the entire system. Finally, for all-atom production runs employing Langevin dynamics, we used a constant temperature of 310 K and constant pressure of 1 atm with 2 fs time steps.

The carbonyl oxygen–Ca^2+^ interaction energy is underestimated in the CHARMM27 force field^41^. Thus, we repeated the Ca^2+^-bound simulation with the G45O-Ca^2+^ distance restrained to 2.35 Å, with a spring constant of 40 kcal mol^−1^.

PMF values were calculated based on the K^+^ and Cl^−^ ion density distributions along the Ca^2+^-bound and Ca^2+^-free Cx26 GJC pore (the *Z* axes) during 30 ns of the all-atom MD simulation production phases (Supplementary Fig. 10). Ions (either K^+^ or Cl^−^) were counted at each position along the *Z* axis with a fixed radius equal to the minimum channel radius (7 Å, at K41) and a Δ*Z* of 5 Å. Ion densities (<*ρ*(*Z*)>) were calculated by dividing the total number of ions at each position on *Z* axis by the corresponding cylindrical volume. PMFs were ultimately calculated by solving the equation,


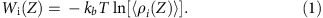


PMF values were adjusted so that values in bulk solvent were zero^64^.

### Electrostatic surface calculations by Internal Coordinates Mechanics and the REBEL method

Electrostatic surface potentials of the Ca^2+^-bound and Ca^2+^-free Cx26 GJCs were calculated by the Rapid Exact-Boundary Electrostatics (REBEL) method^65^ as implemented in the Molsoft software package, ICM-Pro^66^. The REBEL calculations employ a model in which the solvent is a continuous high dielectric medium and the protein molecular surface is the boundary between the solvent and the low dielectric protein. The electrostatic energy is calculated for a set of point charges on the protein surface by solving the Poisson equation (-∇(*ɛ* (**r**)∇*φ* (**r**))=*ρ* (**r**)), where *ɛ* is the dielectric constant, *φ* is the electric potential and *ρ* is the surface charge density. REBEL uses the boundary element method to solve the Poisson equation for the model system described above, where the boundary is the triangulated analytical molecular surface as described by Connolly^67^. A limitation of the boundary element method is that a computationally expensive integral equation solution is required to determine the charge distribution at the boundary. To make the calculations tractable, the molecular surface is divided into discrete elements. REBEL uses a simplified version of the multigrid boundary element discretization method established by Vorobjev and Scheraga^68^, in which the number of boundary elements is reduced by combining all of the elements associated with each surface atom. Consequently, the number of composite boundary elements is reduced to the number of surface atoms without a significant loss in the precision of the charge density calculation^65^.

The parameters for the REBEL boundary element electrostatic calculations were as follows. Partial charges for atoms of amino acids were assigned according to the molecular mechanics force-field named Empirical Conformational Energy Program for Peptides (ECEPP)^69^; glutamate and aspartate were assigned a unitary negative charge and arginine and lysine were assigned a unitary positive charge. The atomic radii used in the electrostatic and surface calculations were: carbon, 1.700 Å; nitrogen, 1.447 Å, nitrogen in nitro groups, 1.763 Å; oxygen in carbonyl, 1.542 Å; oxygen in hydroxyls, 1.500 Å; sulfur in disulfide bonds, 1.491 Å, and sulfur in thiols, 1.812 Å, calcium ion, 1.650 Å. The dielectric constants inside and outside the protein (bulk solvent) were 4 and 78.5, respectively. The patch size for which individual surface elements were assumed to have the same polarized charge surface density in the REBEL boundary element method was set to 0.5 Å.

In the case of Ca^2+^-bound Cx26, we performed REBEL calculations with and without the explicit inclusion of the waters that were found to complete Ca^2+^-coordination in the MD simulations, and we found no significant difference in the resultant electrostatic surface potentials.

Electrostatic surface potentials were also calculated using Poisson–Boltzmann equations as implemented in Adaptive Poisson-Boltzmann Solver (APBS). Surfaces calculated in this way are essentially identical to surfaces calculated with the REBEL method implemented in ICM-Pro.

### Sequence analysis and alignment for determining residue propensity

**Figure 5:**
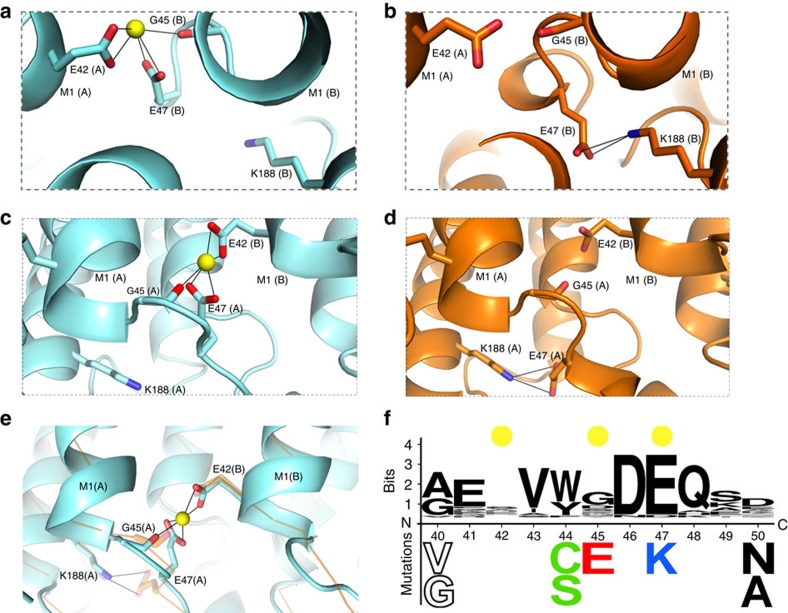
Differences between the Ca^2+^-bound and Ca^2+^-free structures are localized to the Ca^2+^-binding sites. The Ca^2+^-bound (cyan) and Ca^2+^-free (orange) structures, as well as the Ca^2+^ ions (yellow) are coloured identically to Figs 2, 3, 4. (**a**,**c**) Close-up cytoplasmic and side views, respectively, of the Ca^2+^ site in the Ca^2+^-bound structure, corresponding to the dashed boxes in **b** and **d** of Fig. 3. (**b**,**d**) Close-up cytoplasmic and side views, respectively, of the Ca^2+^ site in the Ca^2+^-free structure, corresponding to the dashed boxes in **f** and **h** of Fig. 3. (**e**) Superposition of the Ca^2+^-free structure (orange) onto the Ca^2+^-bound structure (cyan). The E47 side chain adopts different rotamer configurations in the two structures, with the carboxylate stabilized by an ionic interaction with the terminal amine of the K188 side chain in the Ca^2+^-free structure. The E47 side chain adopts different rotamer configurations in the two structures, with the carboxylate stabilized by an ionic interaction with the terminal amine of the K188 side chain in the Ca^2+^-free structure (**b**,**d**). (**f**) Sequence propensity diagram of all 21 human Cx isoforms spanning residues 40–50 that include the Cx26 Ca^2+^-binding residues; this is also a locus harbouring several Cx26 deafness mutations (A40, W44, G45, D46, E47 and D50). Residue numbering is based on the Cx26 sequence. Yellow circles indicate the positions of the residues that coordinate Ca^2+^, and deafness-causing mutations are shown below the residue number.

**Figure 6:**
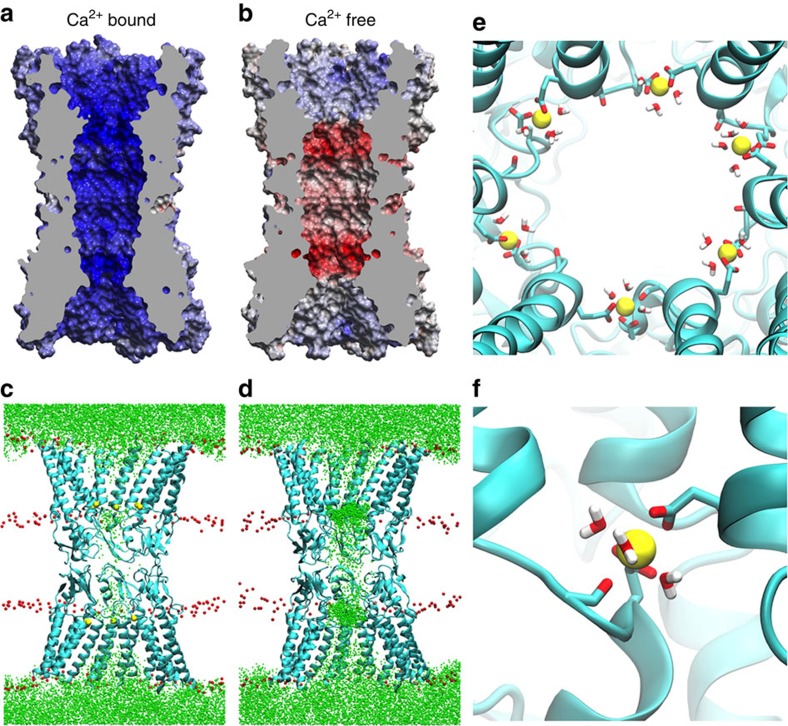
Ca^2+^ binding functions as an electrostatic switch that dramatically restricts K^+^ permeation. Depictions of GJC electrostatic surfaces (**a**,**b**) and molecular dynamics (MD) simulations (**c**,**d**). Comparison of Ca^2+^-bound (**a**,**c**) and Ca^2+^-free (**b**,**d**) states suggests that Ca^2+^ binding creates a positive surface potential in the pore (blue) that restricts cation permeation. Electrostatic potential surfaces (**a**,**b**) with positive and negative electrostatic potentials shown in blue and red, respectively (colour scale is −15 to +15 kT e^−1^). The protein interior is grey. Cross-sectional side views for (**a**) Ca^2+^-bound and (**b**) Ca^2+^-free GJCs, coloured according to electrostatic potential. (**c**,**d**) Superposition of K^+^ ion positions (green dots) every 0.1 ns for a 30-ns continuous segment within a 50-ns all-atom MD simulation. Ions are coloured as in Fig. 1b. Cl^−^ ions are not shown for clarity, but a notable observation of the MD simulations is an enhanced occupancy of Cl^−^ ions in the Ca^2+^-bound pore compared with the Ca^2+^-free pore (Supplementary Movie 3). (**e**,**f**) Hemispheric coordination of Ca^2+^ ions by the carboxylate of E47 and the carbonyl oxygen of G45 in one subunit and the carboxylate of E42 in the adjacent subunit enables the binding of water molecules on the luminal side of the channel. Two to three water molecules are bound to each Ca^2+^ ion throughout the MD simulation. The water molecules are shown at a single MD time step.

Homologous sequences from residues 40–50 (Cx26 numbering) for all 21 human Cx isoforms were defined by a sequence alignment performed with PRALINE^70^ and were uploaded to the Weblogo online server (http://weblogo.berkeley.edu) to generate the diagram shown in Fig. 5f.

## Additional information

**Accession codes:** Coordinates and structure factor files for Ca^2+^-bound and Ca^2+^-free Cx26 have been deposited in with the Protein Data Bank under PDB ID 5ER7 and 5ERA, respectively.

**How to cite this article:** Bennett, B. C. *et al*. An electrostatic mechanism for Ca^2+^-mediated regulation of gap junction channels. *Nat. Commun*. 7:8770 doi: 10.1038/ncomms9770 (2016).

## Supplementary Material

Supplementary Information Supplementary Figures 1-10, Supplementary Tables 1-2, Supplementary Discussion and Supplementary References

Supplementary Movie 1 Ca2+ coordination sites reside at the interface between adjacent subunits, near the entrance to the extracellular gap, where local conformational rearrangements enable Ca2+-chelation. Each of the 12 Ca2+ binding sites is comprised of the G45-O and the E47 carboxylate from one subunit, and the E42 carboxylate of the adjacent subunit. The Ca2+-bound and Ca2+-free structures were superimposed, and the morph conformations tool in Chimera was used to create a conformational trajectory between the two structures (morph model, cyan; Ca2+ atom, yellow; coordinating atoms, sticks).

Supplementary Movie 2 A view showing the transition between the Ca2+-bound and Ca2+-free gap junction channels, highlighting the E47:K188 salt bridge in the Ca2+-free structure. Supplementary Movie 1 is shown at an oblique angle to emphasize the large movement of the E47 side chain, which swings > 90o and 7 Å away from the Ca2+ coordinating conformation to form an intersubunit salt bridge with K188 in the Ca2+-free structure.

Supplementary Movie 3 Molecular dynamics (MD) simulation of a Ca2+-bound gap junction channel shows that the Ca2+ ions induce a strongly positive electrostatic potential that dramatically restricts K+ permeation. Six Ca2+ ions (yellow) bind at the interface between adjacent subunit within the hemichannels that dock end-to-end to form a gap junction channel. All atom MD simulations were performed on the Cx26 X-ray structure (cyan) embedded in two POPC bilayers (phosphorous atoms, red spheres). The cytoplasm and extracellular spaces were ionized with 140 mM KCl (K+, green and Cl-, purple) and 140 mM NaCl (Na+, orange), respectively. (For the purposes of electroneutrality, the Cl-concentration was set to 140 mM. However, we realize that the physiologic cytoplasmic concentration of Cl- is ~4 mM, with most of the additional negative charge contributed by cytoplasmic proteins.) Water molecules are shown as blue lines.

Supplementary Movie 4 Ca2+ ions remain stably bound throughout the MD simulation, with minimal movement of the protein backbone. The E47 carboxylate from one subunit and the E42 carboxylate of the adjacent subunit are stably coordinated throughout the MD simulation, as are three water molecules (red and white sticks). However, coordination by G45-O was only maintained at 1 out of 12 sites in the unrestrained simulation. (Binding site residues shown as sticks; O atoms, red.)

Supplementary Movie 5 The Ca2+ coordination geometry determined by X-ray crystallography is maintained throughout the MD simulation at one of the binding sites. Coordination by G45-O was maintained at this particular site. Restrained MD simulations also maintained coordination by G45-O at every binding site. The electrostatic properties of the channel were not influenced in either the restrained or unrestrained MD simulations. Stick representations and colors are as in Supplementary Movie 4.

Supplementary Movie 6 MD simulation of the Ca2+-free gap junction channel shows that the channel is highly permeable to K+ ions. During the MD simulation, K+ and Cl- ions enter and traverse the channel, and K+ ions cluster in the vicinity of anionic residues at the Ca2+ binding sites.
