# Supplementary material for: An electrostatic mechanism for Ca2+-mediated regulation of gap junction channels
Source: Nat Commun. 2016 Jan 12;7:8770. doi: 10.1038/ncomms9770 (PMC4730032; doi:10.1038/ncomms9770)
Supplement: Supplementary Information — Supplementary Figures 1-10, Supplementary Tables 1-2, Supplementary Discussion and Supplementary References [file ncomms9770-s1.pdf]

# Supplementary Figures

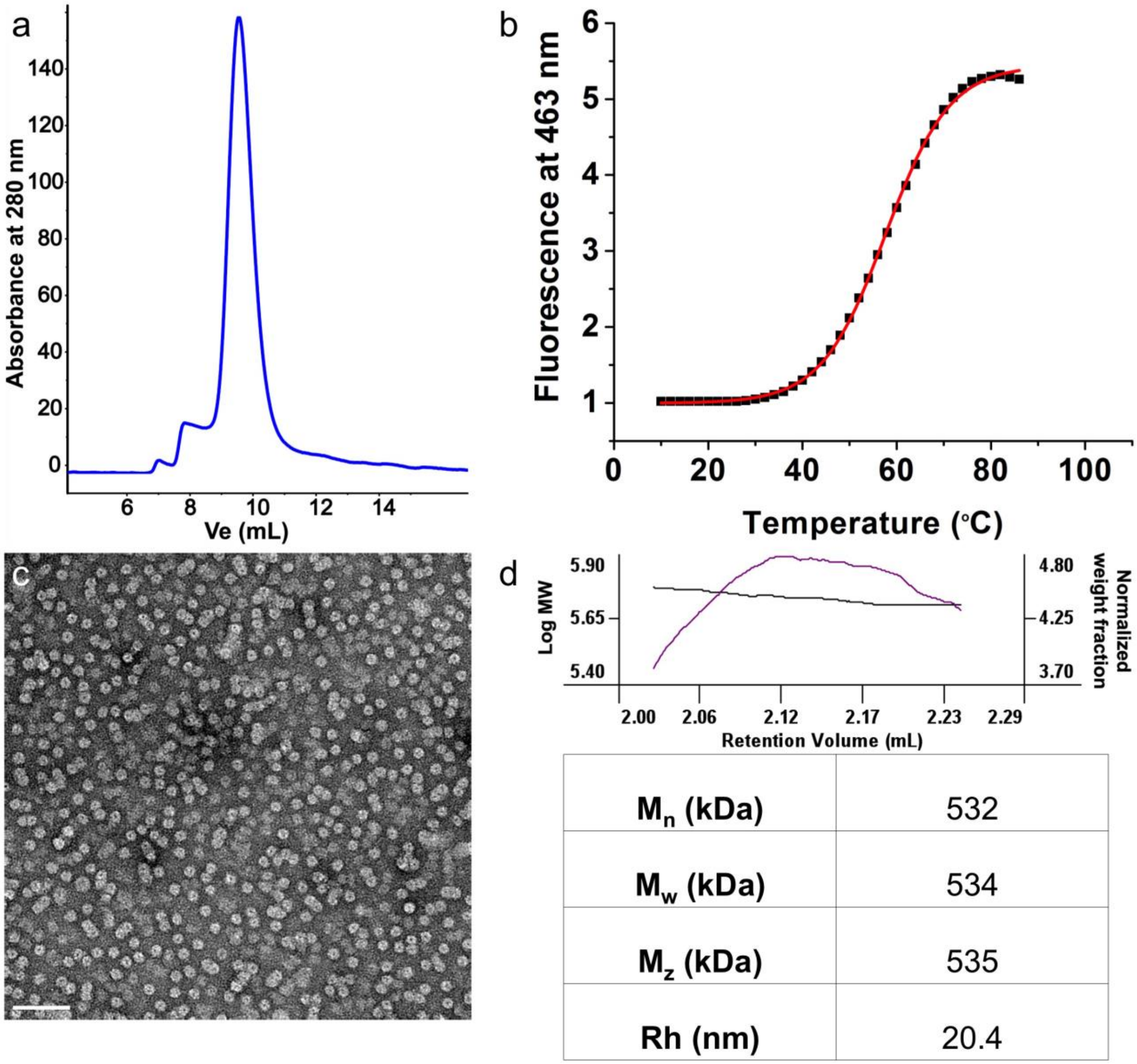


| | |
|---|---|
| $M_n$ (kDa) | 532 |
| $M_w$ (kDa) | 534 |
| $M_z$ (kDa) | 535 |
| Rh (nm) | 20.4 |

**Supplementary Figure 1. Expression, purification and characterization of human Cx26 derived from *Sf9* insect cells.** (**a**) Analytical size-exclusion chromatography (SEC) of Cx26 in a novel facial amphiphile detergent (FA-3) reveals that Cx26 elutes as a dodecamer. The apex of the major peak corresponds to a molecular weight (MW) of 448 kDa, and the calculated MW of a Cx26 dodecamer is ~325 kDa. We attribute the difference between the experimental and calculated MW to the FA-3 micelle mass in the protein:detergent complex (PDC). (**b**) Thermal stability of Cx26 in FA-3 as measured by the fluorescence of a cysteine-reactive, coumarin-based dye (CPM) over a temperature gradient (10 to 85°C)[1]. The Cx26 construct used for crystallization has only one free cysteine at position 202, in the middle of M4 (the other 2 free cysteines, at 211 and 218, were mutated to serine) (see **Fig. 1a**). Upon

increasing temperature, Cx26 unfolds and the dye binds to the C202 thiol and emits fluorescence at 463 nm. The $T_m$ calculated from the Boltzmann fit of the curve is 57.7 °C. (**c**) Electron micrograph of negatively-stained Cx26 in FA-3 showing a field of uniform particles with the characteristic "end-on" view and a central, stain-filled pore. Scale bar, 50 nm. (**d**) Analytical SEC coupled to a quad detector capable of measuring UV absorbance, refractive index (RI), and multiple angle light scattering (MALS) angles of eluted species shows a fairly homogenous population of Cx26:FA-3 PDCs with a median size of 534 kDa. This suggests that ~210 kDa of the PDC can be attributed to the detergent micelle. Note the difference in MW values derived from analytical SEC coupled to just a UV detector compared to analytical SEC with a quad detector system. The latter, employing LS detection, provides a more accurate MW estimate for membrane proteins in detergent because the scattering intensity is directly proportional to the MW of the protein and is independent of shape[2]. The additional detectors for UV and RI can be used to determine the contribution of bound detergent to the MW. The distribution of the molecular mass and size of the PDC is described by several parameters and directly determined in the MALS experiment: $M_n$ is the number average molecular mass and is the arithmetic mean of the mass distribution, $M_w$ is the weight-averaged molecular mass, $M_z$ is the Z-number average molecular mass, and $R_h$ is the hydrodynamic radius of the PDC. Methodological details of the experiments shown in this figure are very similar to those found in our previous work[3].

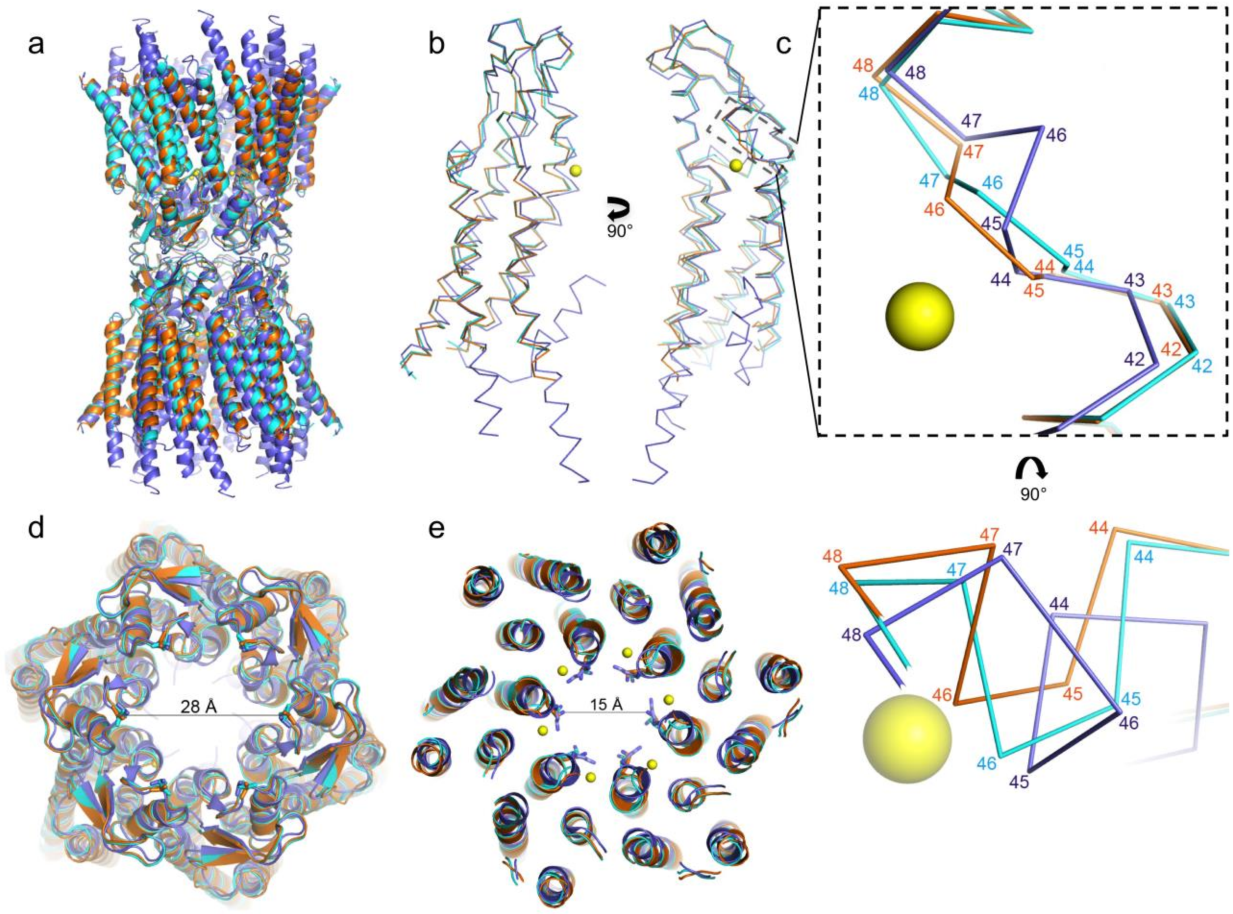


**Supplementary Figure 2. Comparison of $Ca^{2+}$-bound, $Ca^{2+}$-free, and 2ZW3 Cx26 gap junction channels.** Superposition of side views of the (**a**) GJC structures (represented in cartoon) and of (**b**) a single Cx subunit (represented in ribbon). In all panels, the $Ca^{2+}$-bound model is shown in cyan, the $Ca^{2+}$-free model in orange, and the 2ZW3 model in purple, with $Ca^{2+}$ ions shown as yellow spheres. Though the Cα RMSD of the TM α-helices is modest (0.9 Å) and the pore dimensions are very similar, the largest divergence is found in the E1 and E2 loops, with maximum RMSDs of ~4 Å for the Cα atoms and ~8 Å for the side chains (averaged all-atom RMSD is 1.0 Å and 1.3 Å for the E1 and E2 loops, respectively). Specifically, there are two regions of significant backbone deviation between the models reported here and 2ZW3. One occurs near the $Ca^{2+}$-binding site, at residues 42-46 (dashed box) (the other is residues 56-59, as described below). (**c**) A close-up of the dashed-box region from the overlay in (**b**) as viewed (top) from the pore or (bottom) from the extracellular gap. The Cα backbone is shown in ribbon representation, and the average main chain RMSD is 2.6 Å. Residue numbering is shown at the respective Cα atoms for each model. Although there are slight backbone differences between our $Ca^{2+}$-bound and $Ca^{2+}$-free

structures in and around this region (mainly for residues 45-47), the average Cα RMSD is only 0.4 Å, substantially less than the deviation between the $Ca^{2+}$-bound or $Ca^{2+}$-free structures and 2ZW3. In addition, since this sequence forms part of the $Ca^{2+}$ binding site that we identified here and because there was a major side chain reconfiguration at E47, one expects main chain divergence in this region between the $Ca^{2+}$-bound and $Ca^{2+}$-free structures. (**d**) Overlay of $Ca^{2+}$-bound, $Ca^{2+}$-free, and 2ZW3 structures, viewed from the extracellular gap. Residues 56-59 in E1 show moderate backbone deviations between our structures and 2ZW3, with an average Cα RMSD of 1.6 Å (the Cα RMSD for the $Ca^{2+}$-bound and $Ca^{2+}$-free structures for this region is only 0.4 Å). In our structures the backbone bends inwards towards the pore and slightly down along the pore axis. L56 is at the apex of E1 at the hemichannel-docking interface and is shown as sticks. For the 2ZW3 model that was equilibrated via MD[4], the apices of E1 at L56 turn inwards toward the pore. This creates a restriction at the hemichannel-docking interface not observed in any of the crystal structures determined so far. Perhaps this region of E1 may have greater conformational options in undocked hemichannels. (**e**) Overlay of the $Ca^{2+}$-bound (cyan), $Ca^{2+}$-free (orange) and 2ZW3 (purple) structures, viewed from the extracellular gap and shown at the level of the minimum pore diameter within the hemichannel, which is ~15Å between Lys41 residues. The side chains for the Lys41 residues are shown as sticks, with the minimum distance between Nζ atoms shown as a solid line.

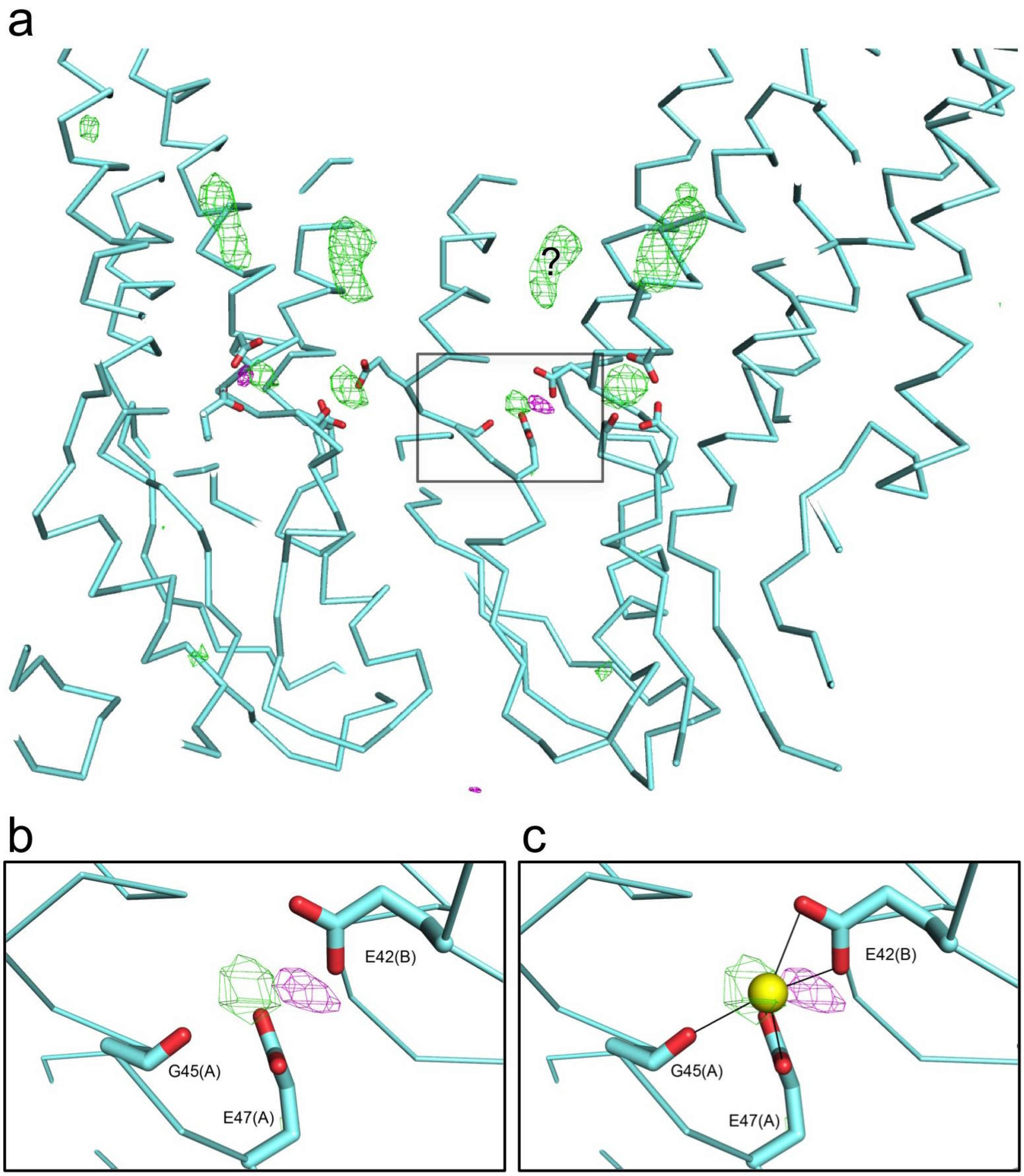


**Supplementary Figure 3. Overlap of difference and anomalous density peaks overlap at the $Ca^{2+}$ binding site.** (**a**) A cross-sectional side view of the hemichannel showing the $Ca^{2+}$ binding sites. The three subunits closest to the viewer have been removed for clarity. $F_o - F_c$ maps are contoured at +4-σ (green mesh), with anomalous maps (magenta mesh) contoured at +5-σ. The "?" designates an unidentified $F_o - F_c$ peak. (**b**) Close-up view of (**a**) showing the difference and anomalous peaks indicative of a bound $Ca^{2+}$ ion. (**c**) The same view as in (**b**) showing

the position of the $Ca^{2+}$ (yellow sphere) after modeling and restrained refinement.

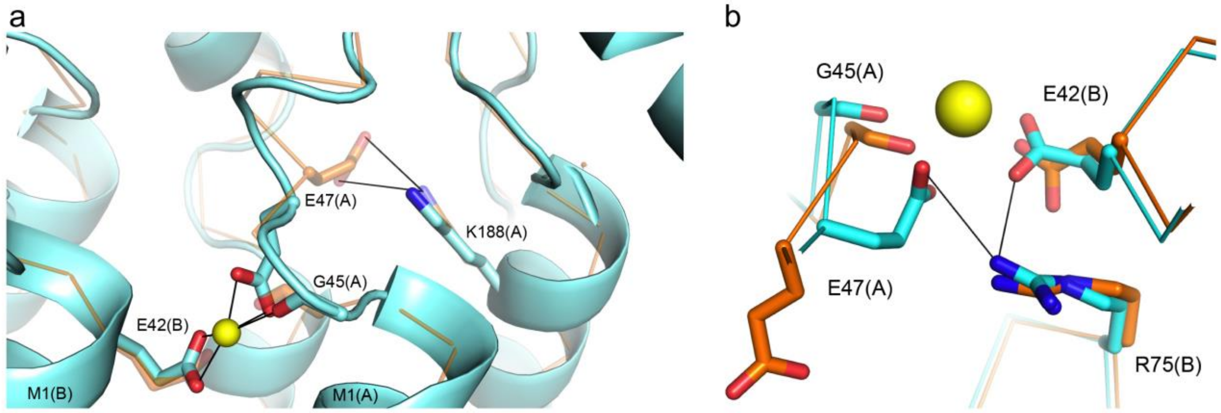


**Supplementary Figure 4. Comparison of $Ca^{2+}$-bound and $Ca^{2+}$-free Cx26 GJCs.** (**a**) Superposition of the (cyan) $Ca^{2+}$-bound and (orange) $Ca^{2+}$-free structures. This is a similar cytoplasmic view for the superposition shown in **Fig. 5E**, except there is a 25º counter-clockwise rotation about the y-axis. Side chains and the G45 carbonyl are shown as semi-transparent thick sticks for the $Ca^{2+}$-free structure, whereas they are shown as solid thin sticks for the $Ca^{2+}$-bound structure. (**b**) A close-up, extracellular view of the $Ca^{2+}$ binding site for the overlay in (**a**). Note the vdW contact and hydrogen bond that forms between R75 and the $Ca^{2+}$-coordinating E42 and E47 residues in the $Ca^{2+}$-bound structure, which is absent in the $Ca^{2+}$-free structures. A notable difference between the backbones of the $Ca^{2+}$-bound and $Ca^{2+}$-free structures is between Trp44 and Gln48, where the $Ca^{2+}$-bound main chain shifts an average of 0.8 Å towards the pore center and away from M1 of the adjacent subunit (see **Supplementary Figure 2C**). The Cα backbone is shown in ribbon representation, and the side chains are shown as sticks. For both panels, the $Ca^{2+}$ ions are shown as yellow spheres.

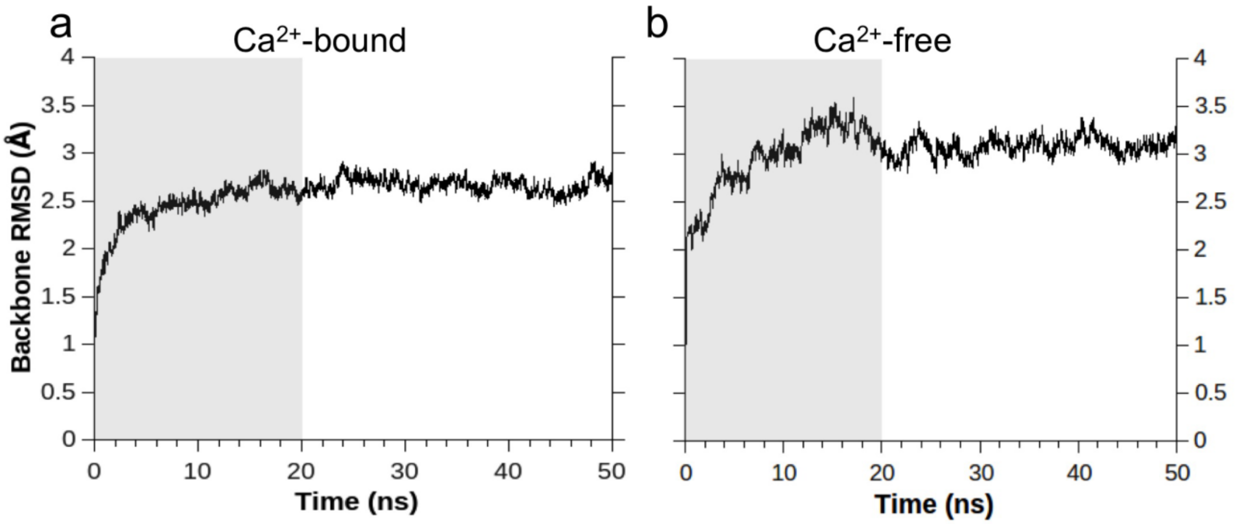


**Supplementary Figure 5. Backbone atom RMS deviations of the Cx26 GJC during MD system equilibration and production runs.** Average deviation (RMSD) of the Cα backbone from the starting model position for the $Ca^{2+}$-bound (**a**) and $Ca^{2+}$-free (**b**) structures over the course of the MD simulations. The shaded area (0-20 ns) corresponds to the equilibration phase of the simulation. The most significant deviations occur in the first few ns of this phase, with the structures remaining relatively stable during the production phase from 20-50 ns.

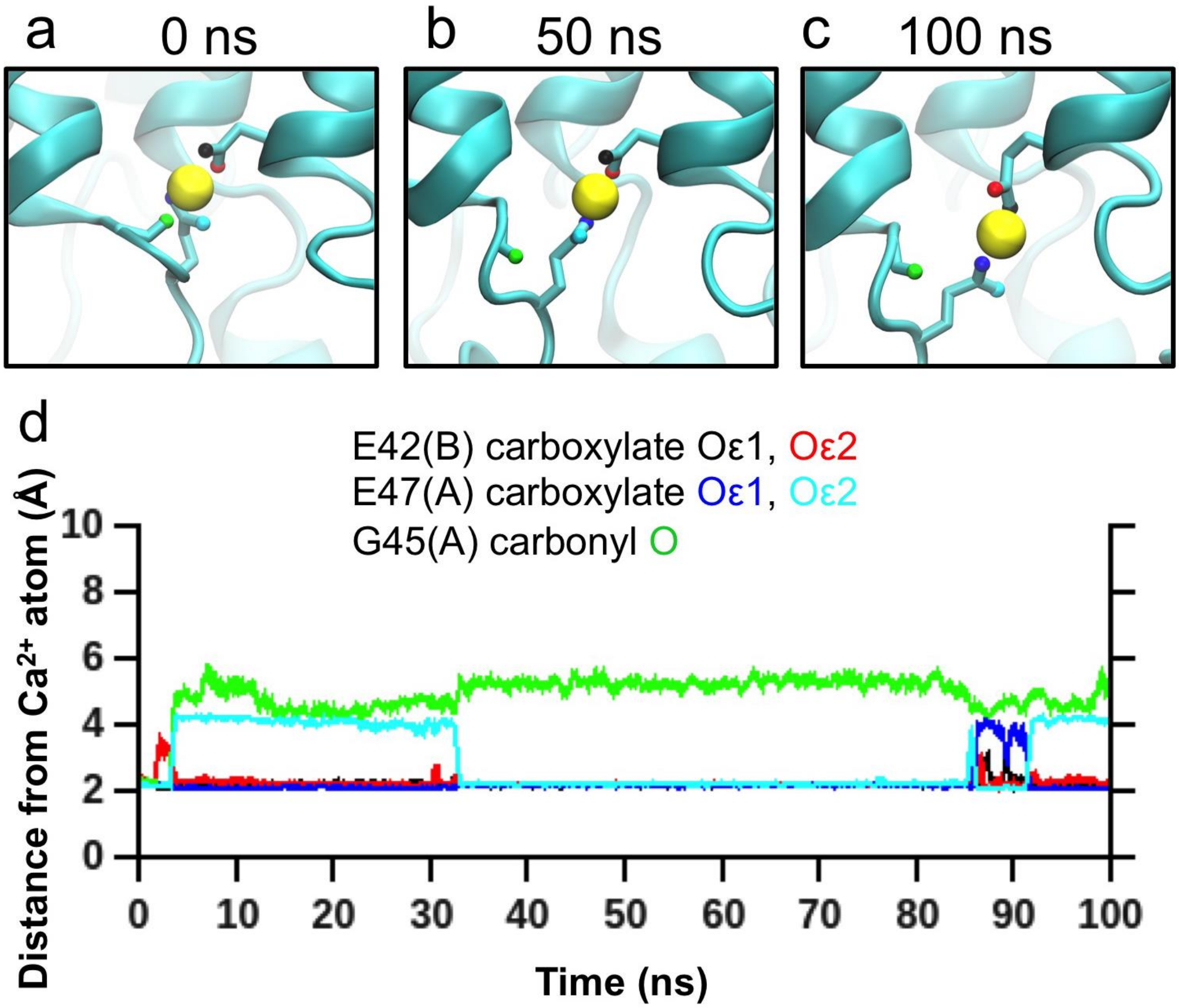


**Supplementary Figure 6. $Ca^{2+}$ coordination distances for the protein ligand atoms in the $Ca^{2+}$-bound GJCs along the MD simulation time course at one binding site.** Changes in the position of $Ca^{2+}$ coordinating atoms are shown at 0, 50 and 100 ns time points within the MD simulation (**a**,**b**,**c**, respectively). (**d**) Changes in the distance (Å) between coordinating atoms and the $Ca^{2+}$ at one of the $Ca^{2+}$ binding sites, plotted over the simulation time course. Atom coloring is indicated in the figure.

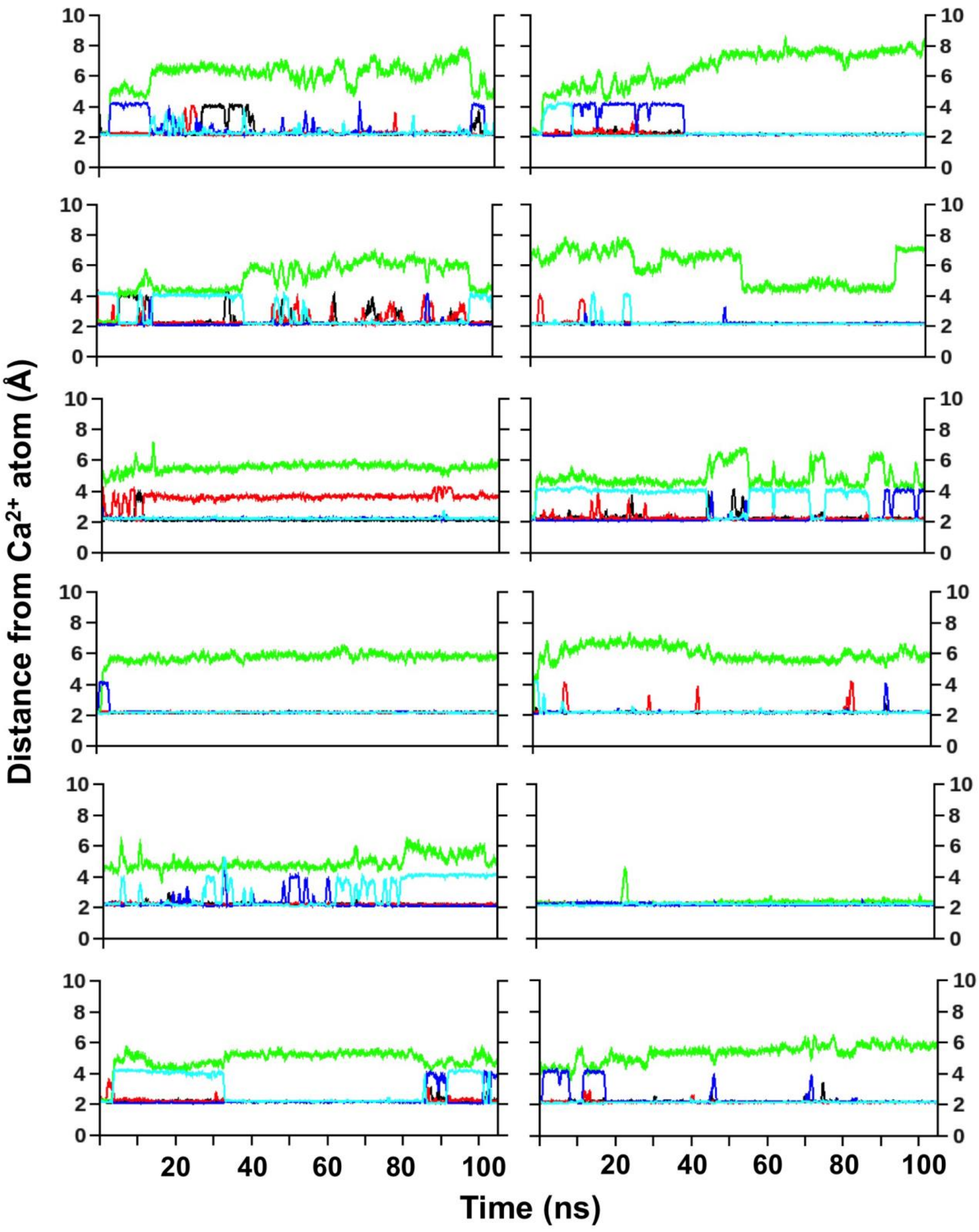


**Supplementary Figure 7. The 12 $Ca^{2+}$ coordination distances for the protein ligand atoms in the $Ca^{2+}$-bound Cx26 GJC along the MD simulation time course.** Distance plots for all 12 $Ca^{2+}$ binding sites in the GJC are

shown. The magnified plot in **Supplementary Figure 6d** corresponds to the plot at the bottom of the left-hand column. The axes are identical to those shown in the magnified plot. Atom coloring is as follows: black – E42 Oε1 (chain B), red – E42 Oε2 (chain B), green – G45 O (chain A), blue – E47 Oε1 (chain A), and cyan – E47 Oε2 (chain A).

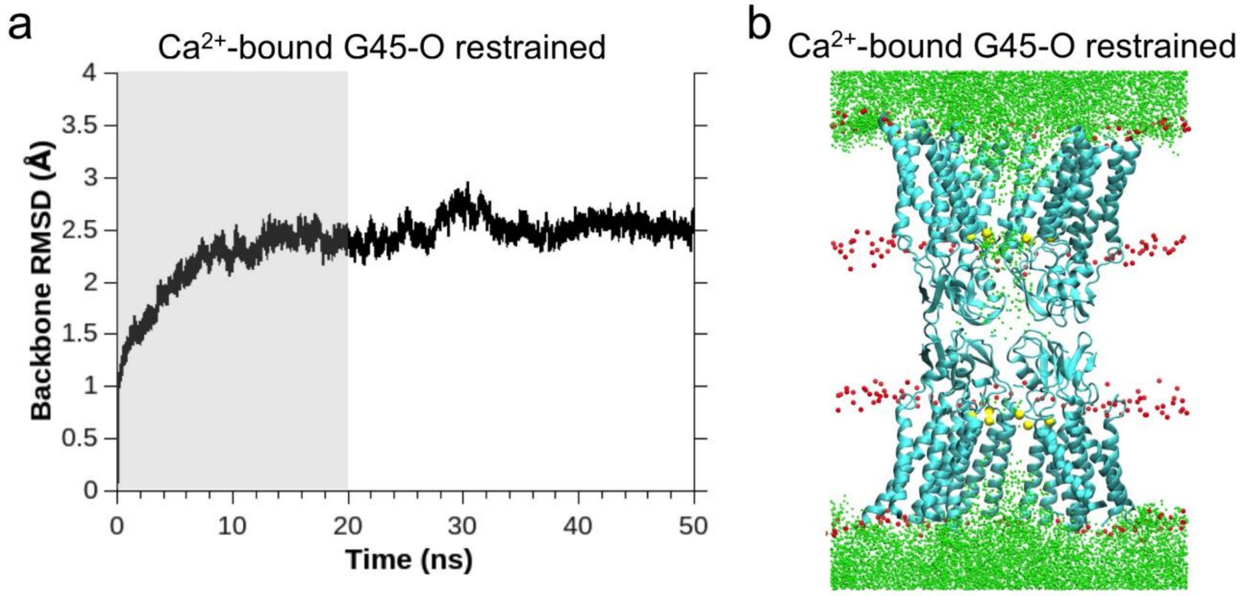


**Supplementary Figure 8. Restraining the G45-O coordination geometry during the MD simulation does not alter the behavior of the $Ca^{2+}$-bound Cx26 GJC.** The G45O-$Ca^{2+}$ distance was restrained to 2.35 Å, with a spring constant of 40 kcal/mol. (**a**) Average RMSD of the Cα backbone from the starting model position over the course of the MD simulation. Plot representation is as in **Supplementary Figure 5**. (**b**) Superposition of $K^+$ ion positions every 0.04 ns for a 30 ns continuous segment within a 50 ns all-atom MD simulation. Ions are colored as in **Fig. 1b,c**.

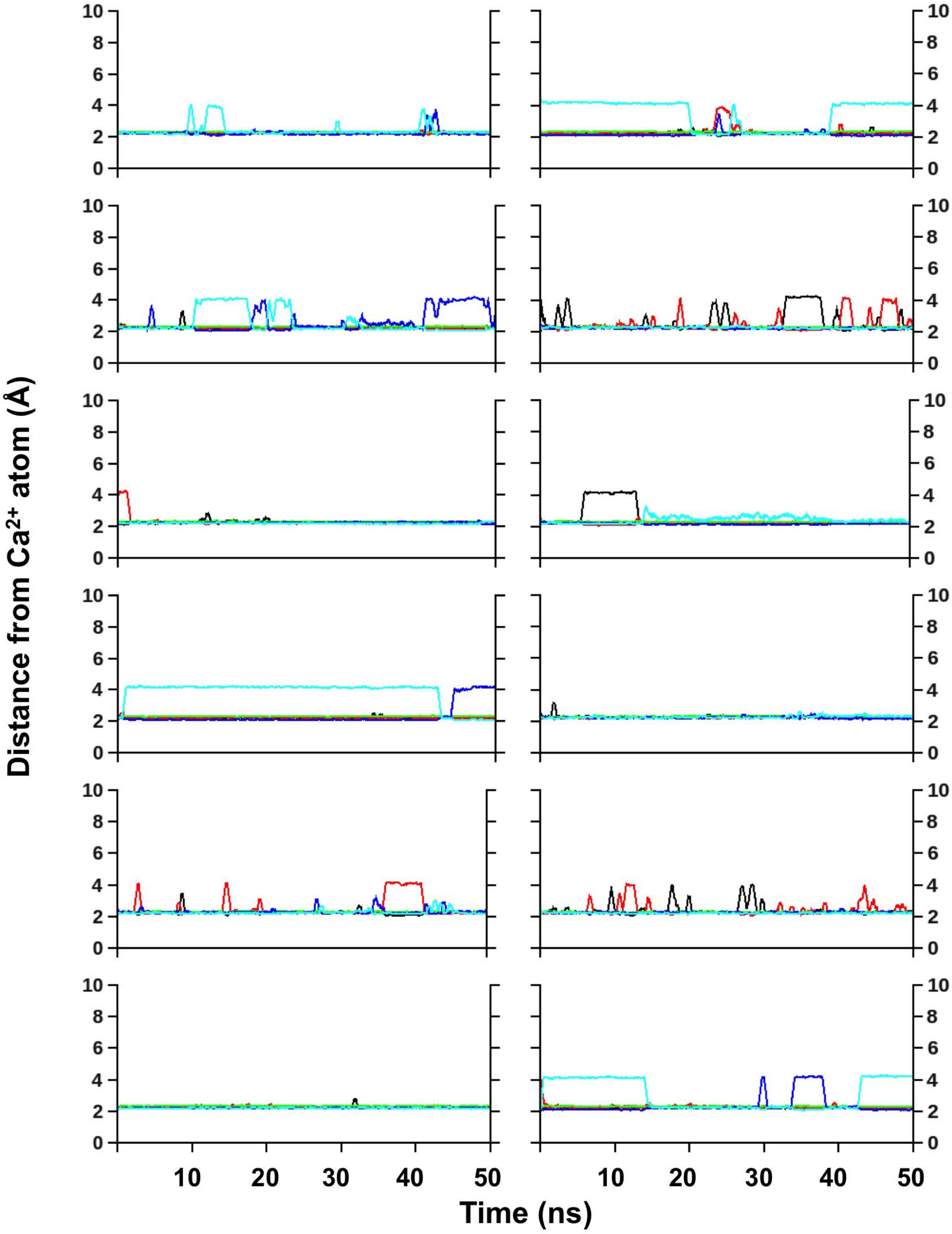


**Supplementary Figure 9. The 12 $Ca^{2+}$ coordination distances for the protein ligand atoms in the $Ca^{2+}$-bound**

**Cx26 GJC along the MD simulation time course when a restraint is added for the coordination distance between G45-O and $Ca^{2+}$.** Distance plots for all 12 $Ca^{2+}$ binding sites in the GJC are shown. The axes and atom coloring are as in **Supplementary Figure 7**. Note the maintenance of coordination by the G45-O at all sites and compare to the distances shown in **Supplementary Figure 7**.

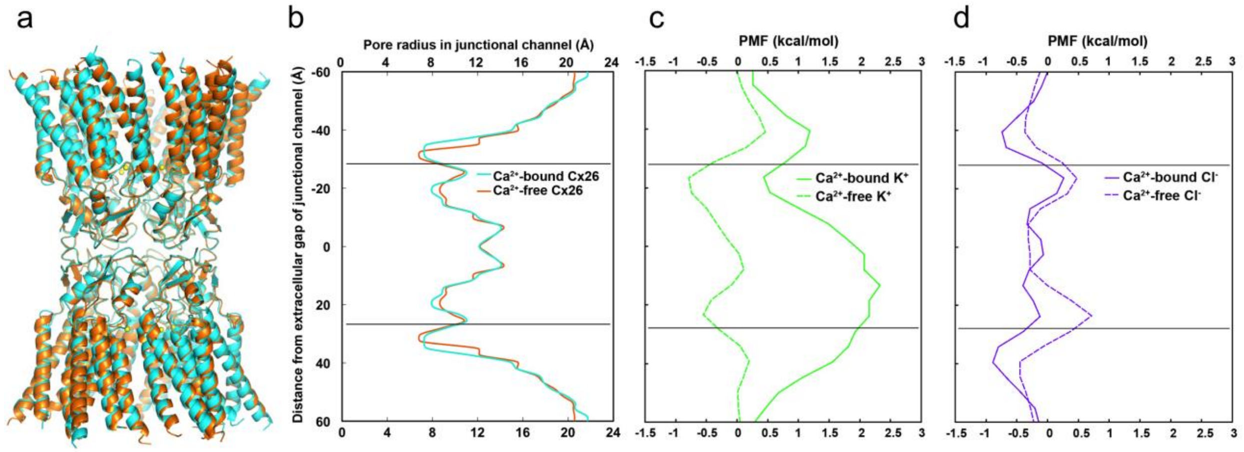


**Supplementary Figure 10. Potential of mean force (PMF) calculations for $K^+$ and $Cl^-$ from all-atom simulations of Cx26 $Ca^{2+}$-bound and $Ca^{2+}$-free GJCs.** (**a**) Superposition of side views of the $Ca^{2+}$-bound (cyan) and $Ca^{2+}$-free (orange) GJC structures. (**b**) Plots of distance from the center of the extracellular gap versus pore radius for the $Ca^{2+}$-bound (cyan) and the $Ca^{2+}$-free channel (orange). For panels **b-d**, the solid horizontal lines indicate the approximate locations of the $Ca^{2+}$ binding sites in their respective hemichannels. In the absence of bound $Ca^{2+}$ (dotted curves, **c-d**), there is a slightly attractive potential for $K^+$ (**c**) and a slightly repulsive potential for $Cl^-$ (**d**) within the pore in the regions of the $Ca^{2+}$ binding sites. Though the pore structure is nearly identical in $Ca^{2+}$-bound and $Ca^{2+}$-free channels (**a-b**), upon $Ca^{2+}$ binding (solid curves, **c-d**) a large repulsive potential for $K^+$ is established throughout the pore (**c**) with a slightly attractive potential for $Cl^-$ (**d**). PMF calculations were based on the $K^+$ and $Cl^-$ ion density distributions along the $Ca^{2+}$-bound and $Ca^{2+}$-free Cx26 GJC axes during 30 ns of the all-atom MD simulation production phases. In the case of the $Ca^{2+}$-bound simulation, a single $K^+$ ion entered the pore and passed the $Ca^{2+}$ binding sites of one hemichannel, where it became trapped (**Fig. 6c** upper hemichannel). The presence of this trapped $K^+$ ion is responsible for the reduced $K^+$ PMF in the vicinity of the upper $Ca^{2+}$ binding site (at ~-30Å in (**c**)), and reflects the limitations of performing PMF calculations on relatively short simulations.

# Supplementary Tables

**Supplementary Table 1: X-ray crystallographic data processing, structure refinement, and model validation statistics.**

| **Crystal Form** | **$Ca^{2+}$-free** | **$Ca^{2+}$-bound** |
|---|---|---|
| Beam line | GM/CA-CAT APS-23-ID | SER-CAT APS-22-ID |
| Space Group | H32 | H32 |
| Cell Dimensions | 155.85, 155.85, 160.80 Å | 156.09, 156.09, 160.07 Å |
| | 90, 90 120° | 90, 90 120° |
| Wavelength (Å) | 1.033 | 0.976 |
| Resolution range (Å) | 44.16 – 3.59 (3.68 – 3.59) | 39.02 – 3.09 (3.24 – 3.09) |
| Completeness (%)* | 99.7 (68.7) | 98.9 (63.3) |
| Mosaicity range (average) | 0.29-0.56 (0.39) | 0.37-0.53 (0.43) |
| Mean I/σ* | 19.8 (2.2) | 19.1 (1.2) |
| Redundancy* | 7.4 (7.5) | 5.9 (6.0) |
| $CC_{1/2}$*¶ | 0.999 (0.723) | 0.999 (0.209^) |

**Refinement Statistics**

| | | |
|---|---|---|
| Resolution range, Å ($CC_{1/2}$ at cutoff) | 44.0 – 3.8 (0.85) | 34.0 – 3.3 (0.50) |
| Number of Unique Reflections | 7398 | 11481 |
| Completeness (%)* | 97.9 (99.9) | 99.0 (91.8) |
| $R_{work}/R_{free}$§ (Reflections in $R_{free}$ set) | 0.301/0.333 (741) | 0.280/0.313 (549) |
| Number of non-hydrogen atoms | Protein: 2539<br>Ligand: 0<br>Water: 0 | Protein: 2575<br>Ligand: 2<br>Water: 0 |
| Average B-factor ($Å^2$) | 127 | 142; $Ca^{2+}$: 137 |

**Molprobity Analysis**

| | | |
|---|---|---|
| Clashscore (percentile) | 2.56 (100th) | 3.49 (100th) |
| Poor Rotamers (%) | 0 | 1.05 |
| Ramachandran Outliers (%) | 0.65 | 2.27 |
| Ramachandran Favored (%) | 91.53 | 89.00 |
| Cβ Deviations > 0.25 Å | 0 | 0 |
| Molprobity Score (percentile) | 1.54 (100th) | 1.73 (100th) |
| Residues with bad bonds (%) | 0 | 0 |
| Residues with bad angles (%) | 0 | 0 |

*Values corresponding to the highest resolution shell are shown in parantheses

¶Intra-data set Pearson Correlation Coefficient, given by $CC_{1/2}$= Corr(I1, I2)=Corr(y1,y2)=Cov(y1,y2)/$\sqrt{}(\sigma_\tau^2+\sigma_\varepsilon^2)(\sigma_\tau^2+\sigma_\varepsilon^2)$.

¶See ref. 5 for details.

^$CC_{1/2}$ calculated to 3.23 Å

resolution

§$R_{work}$ = Residual error between observed and calculated structure factor amplitudes, using the equation $\Sigma| F_o - F_c |/|F_o|$.

§$R_{free}$ is the identical calculation as $R_{work}$, using 5% of the reflections omitted from refinement for cross-validation.

**Supplementary Table 2: A comparison of the structural differences between defined regions of 2ZW3, $Ca^{2+}$-free and $Ca^{2+}$-bound Cx26.**

| Comparison* | 2ZW3:$Ca^{2+}$-free | 2ZW3:$Ca^{2+}$-bound | $Ca^{2+}$-free:$Ca^{2+}$-bound |
|---|---|---|---|
| All Cα atoms | 1.1; 1.0; 4.2¶ | 1.2; 1.0; 5.4 | 0.5; 0.4; 1.1 |
| All side chain atoms | 2.0; 1.6; 7.3 | 1.9; 1.5; 8.4 | 1.1; 0.8; 8.6 |
| TM Cα atoms | 0.8; 0.8; 2.1 | 0.9; 0.8; 2.4 | 0.4; 0.4; 1.1 |
| TM side chain atoms | 1.8; 1.5; 6.7 | 1.8; 1.4; 6.9 | 0.8; 0.6; 2.8 |
| E1 Cα atoms | 1.4; 1.2; 2.8 | 1.4; 1.3; 1.9 | 0.5; 0.4; 1.5 |
| E1 side chain atoms | 2.0; 1.6; 5.8 | 2.0; 1.5; 4.6 | 1.5; 1.0; 8.3 |
| E1(42-46) Cα atoms | 1.4; 1.2; 2.8 | 1.4; 1.3; 1.9 | 0.8; 0.7; 2.3 |
| E1(42-46) side chain atoms | 2.4; 2.2; 4.4 | 2.7; 2.5; 4.6 | 1.3; 1.1; 3.2 |
| E1(56-59) Cα atoms | 1.5; 1.5; 2.2 | 1.6; 1.6; 2.0 | 0.4; 0.4; 0.5 |
| E1(56-59) side chain atoms | 1.3; 1.3; 1.3 | 1.6; 1.6; 1.8 | 0.9; 0.9; 1.2 |
| E2 Cα atoms | 1.5; 1.3; 3.4 | 1.3; 1.1; 2.5 | 0.6; 0.5; 2.3 |
| E2 side chain atoms | 2.2; 1.8; 7.3 | 1.7; 1.4; 5.6 | 1.3; 1.0; 5.7 |

*Residues 18-95 and 135-215 were used for the structure alignment, and the alignment was performed in SUPERPOSE in CCP4 (ref. 6). In the comparison row, the first model listed was superposed (the “moving” molecule) onto the second model listed (the “reference” molecule).
¶The distances given are in Å and are listed as, in order, the RMS deviation, the average deviation, and the maximum deviation for a particular alignment pair.

## Supplementary Discussion

### The cytoplasmic domains of Cx26

The computational studies that we performed did not consider the roles of the NT, CT and CL in $Ca^{2+}$-induced changes in permeation or gating, as these domains were not resolved in the crystal structures. To the extent that these regions shield, enter or interact with the pore, they would be expected to influence permeation in ways not addressed here. For instance, there is evidence that the NT senses voltage and may physically enter the pore[7-10]. If $Ca^{2+}$ binding alters the voltage profile, the occupancy of the voltage-sensing NT within the pore may also be altered, thereby affecting the pore-blocking effects of the NT. Intriguingly, several disease-causing mutations in the NT of Cx26 result in aberrantly open hemichannels with decreased sensitivity to extracellular $Ca^{2+}$ (ref. [11]), empirically similar to some mutations in and near the identified $Ca^{2+}$ binding sites. This suggests an interaction between the NT and $Ca^{2+}$ sensitive processes, at least in hemichannels (see below), and perhaps in junctional channels as well.

### Justification for independent structure determination

The first X-ray crystallographic structure of the Cx26 gap junction channel (PDB ID: 2ZW3) was solved in a heroic effort by the laboratory of Dr. Tomitake Tsukihara. The challenging and meticulous structure determination has been described in detail[12,13]. The obvious method for phasing our Cx26 X-ray crystallographic data was molecular replacement with the 2ZW3 model, and this is the method we used initially to solve the $Ca^{2+}$-bound Cx26 structure. However, after refinement of the model, there was very weak density for the cytoplasmic extensions of the TM helices and no electron density at the position of the N-terminal helices present in the 2ZW3 model. In an effort to understand the absence of electron density corresponding to the N-terminal and cytoplasmic domains in our maps, we calculated $2F_o - F_c$

and $F_o - F_c$ maps from the deposited 2ZW3 coordinates and structure factor amplitudes. The resultant 2ZW3 maps contained only weak density in the regions of the N-terminal helices. We contacted the authors of the 2ZW3 structure who provided clarification regarding the modeling of the N-terminus and an image of a simulated annealing omit map (but not the map file), which was calculated without the N-terminal domains of the model. The image, a cytoplasmic view of the pore, showed some contiguous electron density at the positions of the N-termini. However, we decided that it was prudent to solve our Cx26 structures without the aid of 2ZW3 in an attempt to resolve with certainty any differences in the structure of the N-terminal domain. Ultimately, we were unable to model the cytoplasmic TM extensions or the N-terminus in either our $Ca^{2+}$-bound or $Ca^{2+}$-free structure.

**Relevance of our results on gap junction channels to hemichannel physiology**

Normal extracellular $Ca^{2+}$ keeps unapposed hemichannels in the plasma membrane in a predominantly closed/non-conductive state. We suspect that the $Ca^{2+}$ binding sites that we identified likely involve some or all of the same residues that regulate hemichannels. The basis for this assertion is that the $Ca^{2+}$ binding sites reside in a region that has been implicated across multiple connexin isoforms as a locus for hemichannel regulation by extracellular divalent cations, based on deafness-causing mutations that result in aberrantly open hemichannels and on experimental mutagenesis[14-20]. In addition, it has been proposed that a network of electrostatic interactions in this region[21] stabilizes a conductive state, and that specific electrostatic interactions are disrupted by extracellular $Ca^{2+}$ to destabilize it[17,18]. Furthermore, conformational changes associated with loop gating, a voltage gating process that can be modulated by divalent cations, map to this locus[22-24]. Altogether, this demonstrates the importance of the M1/E1

segment in the function and regulation of GJCs and hemichannels.

While the same or overlapping residues in GJCs and hemichannels may coordinate $Ca^{2+}$, the structural and functional consequences of $Ca^{2+}$ binding may differ. For instance, it is possible that large-scale $Ca^{2+}$-induced structural changes and/or localized effects on structural and electrostatic interactions in hemichannels are precluded upon docking of hemichannels. Previous AFM imaging of the extracellular surface of hydrated hemichannels showed significant $Ca^{2+}$-induced narrowing of the pore[25]. However, we note that the hemichannels were created by force dissection of intact gap junction plaques, which may disrupt the protein conformation on the extracellular surface. In addition, metal-bridging experiments indicated no significant structural rearrangements and/or narrowing of the extracellular pore during loop-gate closure[4], which involves much of the same segments of the protein. Admittedly, the relation between loop-gating and $Ca^{2+}$ gating is unclear[14], and we realize that discernment of $Ca^{2+}$ effects on Cx hemichannels will require structures at high resolution.

Given these observations and provisos, we are not aware of any electrophysiological studies of wild-type Cx26 hemichannels that show evidence for a $Ca^{2+}$-dependent transition to anion selectivity. Such a transition would be expected if the only effect of $Ca^{2+}$ binding were to decrease cation permeability. In the MD simulations reported here, we did observe residence of $Cl^-$ ions in the $Ca^{2+}$-free channel pore, and this population was enhanced in the $Ca^{2+}$-bound channel. A logical extension from the simulations is that one might expect that the $Ca^{2+}$-bound channel should allow $Cl^-$ conductance. In addition, due to the $Ca^{2+}$-induced positive electrostatic surface potentials, the $Cl^-$ conductance may be greater than what is observed in the $Ca^{2+}$-free channel. We note that in a junctional channel, the magnitude of the $Cl^-$ conductance would be very low due to the relatively low intracellular $Cl^-$ concentration (~4 mM). However, in the case

of hemichannels, the $Cl^-$ conductance might be significant because the extracellular end of the pore faces a milieu that includes ~120 mM $Cl^-$. It is still possible that $Ca^{2+}$ binding to hemichannels results in occlusion of the pore by a physical gate, and state-dependent chemical accessibility studies suggest that this may be the case[22,26]. Of course, physical closure would likely attenuate conductance of all ions including $Cl^-$. The unitary conductance of Cx46 hemichannels is attenuated when $Ca^{2+}$ is applied, even in the presence of voltage[26]. This could be interpreted as $Ca^{2+}$-block of $K^+$ permeability, as proposed here; however, to verify this, further experiments are needed to specifically test this on Cx26 GJCs and hemichannels.

Extracellular $Ca^{2+}$ regulates the activity of unapposed connexin hemichannels, with apparent affinities ranging from 0.15 to 1.3 mM, depending on the connexin (1.3 mM for Cx26)[14,18,27-32]. Open hemichannels formed by Cx26 appear to be highly permeable to $Ca^{2+}$ (refs. [16,33]). This could present a conundrum, since it suggests that the channels are open and permeable to $Ca^{2+}$ at concentrations near millimolar. However, it is possible that there is much greater conformational flexibility in undocked hemichannels versus intact GJCs. Such flexibility may reduce the $Ca^{2+}$ binding affinity relative to that of the junctional channel. It is also possible that conformational flexibility in hemichannels may enable participation of additional nearby residues in $Ca^{2+}$ coordination.

## Relation of our findings to other data on the location of sites for $Ca^{2+}$ binding and regulation

Given the experimental constraints of studying GJCs, all physiological and mutagenesis studies on the mechanism of direct regulation by $Ca^{2+}$ have been performed on unapposed hemichannels. Nearly all of these studies point to the pore-lining region of E1 that includes the

$Ca^{2+}$ coordinating residues identified here (E47, G45, E42), or to the corresponding residues in other connexins, as the region at which $Ca^{2+}$ acts. It is notable that a near-contiguous group of deafness-causing point mutations resides in the $Ca^{2+}$-binding region of Cx26 (**Figs. 1a, 5f**)[34]. We propose that such mutations may alter $Ca^{2+}$ binding and result in channelopathies.

In Cx26 hemichannels, electrophysiological studies combined with site-directed mutagenesis, chemical modification and thermodynamic mutant cycle analysis have identified residues and interactions that are involved in $Ca^{2+}$ sensing and the consequent gating changes. The published data indicate that $Ca^{2+}$ sensitivity has a strong requirement for a negatively charged residue at position 50 (D50 in wild-type channels), which interacts with Q48 and/or K61 in an adjacent subunit in a $Ca^{2+}$ sensitive manner[17,18,35]. These studies do not identify these residues as directly coordinating the $Ca^{2+}$ but make clear that they are intimately involved in the molecular mechanism by which $Ca^{2+}$ gates the channels. Recent MD simulations have also implicated this region as a $Ca^{2+}$ binding site, identifying E42, D46, E47 and D50 as particularly important for coordination[36].

An exception to the focus on residues within E1 as the site for $Ca^{2+}$ binding is the proposal that two aspartate residues (169 and 178) within E2 of adjacent subunits in Cx32 harbor the divalent cation-binding site; this is based on decreased $Ca^{2+}$ sensitivity of voltage dependence when the residues are mutated to asparagine[31]. However, this site could be specific to Cx32 since an anionic residue in the vicinity of position 169 is not present in most connexin isoforms, yet these are well regulated by extracellular $Ca^{2+}$.

## Supplementary References


1 Alexandrov, A. I., Mileni, M., Chien, E. Y., Hanson, M. A. & Stevens, R. C. Microscale fluorescent thermal stability assay for membrane proteins. *Structure* **16**, 351-359, (2008).

2 Korepanova, A. & Matayoshi, E. D. HPLC-SEC characterization of membrane protein-detergent complexes. *Curr Protoc Protein Sci* **Chapter 29**, Unit 29 25 21-12, (2012).

3 Lee, S. C. *et al.* Steroid-based facial amphiphiles for stabilization and crystallization of membrane proteins. *Proc Natl Acad Sci U S A* **110**, E1203-1211 (2013).

4 Kwon, T., Tang, Q. & Bargiello, T. A. Voltage-dependent gating of the Cx32*43E1 hemichannel: conformational changes at the channel entrances. *The Journal of General Physiology* **141**, 243-259 (2013).

5 Karplus, P. A. & Diederichs, K. Linking crystallographic model and data quality. *Science* **336**, 1030-1033 (2012).

6 Collaborative Computational Project, N. The CCP4 Suite: Programs for Protein Crystallography. *Acta Crystallographica* **D50**, 760-763 (1994).

7 Verselis, V. K., Ginter, C. S. & Bargiello, T. A. Opposite voltage gating polarities of two closely related connexins. *Nature* **368**, 348-354 (1994).

8 Oh, S., Rubin, J. B., Bennett, M. V., Verselis, V. K. & Bargiello, T. A. Molecular determinants of electrical rectification of single channel conductance in gap junctions formed by connexins 26 and 32. *J Gen Physiol* **114**, 339-364 (1999).

9 Purnick, P. E., Oh, S., Abrams, C. K., Verselis, V. K. & Bargiello, T. A. Reversal of the gating polarity of gap junctions by negative charge substitutions in the N-terminus of connexin 32. *Biophys J* **79**, 2403-2415 (2000).

10 Harris, A. L. Voltage-sensing and substate rectification: moving parts of connexin channels. *J Gen Physiol* **119**, 165-169 (2002).

11 Lee, J. R., Derosa, A. M. & White, T. W. Connexin mutations causing skin disease and deafness increase hemichannel activity and cell death when expressed in Xenopus oocytes. *J Invest Dermatol* **129**, 870-878 (2009).

12 Maeda, S. *et al.* Structure of the connexin 26 gap junction channel at 3.5 A resolution. *Nature* **458**, 597-602 (2009).

13 Suga, M., Maeda, S., Nakagawa, S., Yamashita, E. & Tsukihara, T. A description of the structural determination procedures of a gap junction channel at 3.5 A resolution. *Acta crystallographica. Section D, Biological crystallography* **65**, 758-766 (2009).

14 Verselis, V. K. & Srinivas, M. Divalent cations regulate connexin hemichannels by modulating intrinsic voltage-dependent gating. *The Journal of General Physiology* **132**, 315-327 (2008).

15 Gerido, D. A., DeRosa, A. M., Richard, G. & White, T. W. Aberrant hemichannel properties of Cx26 mutations causing skin disease and deafness. *American Journal of Physiology Cell Physiology* **293**, C337-345 (2007).

16 Sánchez, H. A., Mese, G., Srinivas, M., White, T. W. & Verselis, V. K. Differentially altered Ca2+ regulation and Ca2+ permeability in Cx26 hemichannels formed by the A40V and G45E mutations that cause keratitis ichthyosis deafness syndrome. *J Gen Physiol* **136**, 47-62 (2010).

17 Sanchez, H. A., Villone, K., Srinivas, M. & Verselis, V. K. The D50N mutation and syndromic deafness: Altered Cx26 hemichannel properties caused by effects on the pore

and intersubunit interactions. *J Gen Physiol* **142**, 3-22 (2013).
18 Lopez, W., Gonzalez, J., Liu, Y., Harris, A. L. & Contreras, J. E. Insights on the mechanisms of Ca2+ regulation of connexin26 hemichannels revealed by human pathogenic mutations (D50N/Y). *J Gen Physiol* **142**, 23-35 (2013).
19 Palacios-Prado, N. *et al.* Intracellular magnesium-dependent modulation of gap junction channels formed by neuronal connexin36. *J Neurosci* **33**, 4741-4753 (2013).
20 Palacios-Prado, N. *et al.* Molecular determinants of magnesium-dependent synaptic plasticity at electrical synapses formed by connexin36. *Nature communications* **5**, 4667, (2014).
21 Kwon, T. *et al.* Molecular dynamics simulations of the Cx26 hemichannel: insights into voltage-dependent loop-gating. *Biophys J* **102**, 1341-1351 (2012).
22 Pfahnl, A. & Dahl, G. Localization of a voltage gate in connexin46 gap junction hemichannels. *Biophysical Journal* **75**, 2323-2331 (1998).
23 Tang, Q., Dowd, T. L., Verselis, V. K. & Bargiello, T. A. Conformational changes in a pore-forming region underlie voltage-dependent "loop gating" of an unapposed connexin hemichannel. *J Gen Physiol* **133**, 555-570 (2009).
24 Verselis, V. K., Trelles, M. P., Rubinos, C., Bargiello, T. A. & Srinivas, M. Loop gating of connexin hemichannels involves movement of pore-lining residues in the first extracellular loop domain. *J Biol Chem* **284**, 4484-4493 (2009).
25 Müller, D. J., Hand, G. M., Engel, A. & Sosinsky, G. E. Conformational changes in surface structures of isolated connexin 26 gap junctions. *The EMBO Journal* **21**, 3598-3607 (2002).
26 Pfahnl, A. & Dahl, G. Gating of cx46 gap junction hemichannels by calcium and voltage. *Pflugers Arch* **437**, 345-353 (1999).
27 Zampighi, G. A., Loo, D. D., Kreman, M., Eskandari, S. & Wright, E. M. Functional and morphological correlates of connexin50 expressed in Xenopus laevis oocytes. *J Gen Physiol* **113**, 507-524 (1999).
28 Beahm, D. L. & Hall, J. E. Hemichannel and junctional properties of connexin 50. *Biophys J* **82**, 2016-2031 (2002).
29 Contreras, J. E., Sáez, J. C., Bukauskas, F. F. & Bennett, M. V. Gating and regulation of connexin 43 (Cx43) hemichannels. *Proc Natl Acad Sci U S A* **100**, 11388-11393 (2003).
30 Ebihara, L., Liu, X. & Pal, J. D. Effect of external magnesium and calcium on human connexin46 hemichannels. *Biophys J* **84**, 277-286 (2003).
31 Gómez-Hernández, J. M., de Miguel, M., Larrosa, B., González, D. & Barrio, L. C. Molecular basis of calcium regulation in connexin-32 hemichannels. *Proc Natl Acad Sci U S A* **100**, 16030-16035 (2003).
32 Fasciani, I. *et al.* Regulation of connexin hemichannel activity by membrane potential and the extracellular calcium in health and disease. *Neuropharmacology* **75**, 479-490 (2013).
33 Fiori, M. C. *et al.* Permeation of calcium through purified connexin 26 hemichannels. *J Biol Chem* **287**, 40826-40834 (2012).
34 Hoang Dinh, E. *et al.* Diverse deafness mechanisms of connexin mutations revealed by studies using in vitro approaches and mouse models. *Brain Research* **1277**, 52-69 (2009).
35 Lopez, W., Liu, Y., Harris, A. L. & Contreras, J. E. Divalent regulation and intersubunit interactions of human Connexin26 (Cx26) hemichannels. *Channels (Austin)* **8** (2013).
36 Zonta, F., Polles, G., Zanotti, G. & Mammano, F. Permeation pathway of homomeric

connexin 26 and connexin 30 channels investigated by molecular dynamics. *J Biomol Struct Dyn* **29**, 985-998 (2012).
